# Brown Algae from San Andres Island, Southwest Caribbean: A Nuclear Magnetic Resonance Spectroscopy–Metabolomic Study

**DOI:** 10.3390/metabo15050305

**Published:** 2025-05-02

**Authors:** Felipe de la Roche, Sara P. Abril, Lady J. Sepulveda, Anderson Piza, Leonardo Castellanos, Natalia Rincón, Mónica Puyana, Freddy A. Ramos

**Affiliations:** 1 Universidad Nacional de Colombia, Facultad de Ciencias, Departamento de Química, Carrera 30 # 45-03, Bogotá 16486, Colombia; fde@unal.edu.co (F.d.l.R.); spabrilp@unal.edu.co (S.P.A.); lysepulvedas@unal.edu.co (L.J.S.); aepizas@unal.edu.co (A.P.); lcastellanosh@unal.edu.co (L.C.); 2 Departamento de Ciencias Biológicas y Ambientales, Universidad Jorge Tadeo Lozano, Carrera 4 # 22-61, Bogotá 111321, Colombia; marthan.rincond@utadeo.edu.co (N.R.); monica.puyana@utadeo.edu.co (M.P.)

**Keywords:** Dictyotales, diterpenes, *Canistrocarpus*, *Stypopodium*, marine natural products

## Abstract

**Background:** Brown algae from the order Dictyotales are known to produce specialized metabolites with a wide array of biological activities. Studying these compounds is important for understanding their ecological roles, exploring biomedical potential and developing biotechnological applications. **Methods:** To evaluate the metabolic diversity of brown algae from the shallow habitats of the northern region of San Andrés Island (Colombia, SW Caribbean), a metabolic profiling approach was employed, based on ^1^H-NMR spectra taken from organic extracts. Four sampling expeditions were conducted to collect the most abundant species, taking into account the taxonomic identity, growth substrate and collection date. **Results:** Five species were found and identified as *Canistrocarpus crispatus*, *Stypopodium zonale*, *Dictyopteris delicatula*, *Padina gymnospora* and *Dictyota* spp. Multivariate analyses applied to these spectra revealed that *S. zonale* and *C. crispatus* differentiated from the other samples mainly due to the signals for meroditerpenes and diterpenes, respectively. *S. zonale* had differential metabolic production observed when comparing algae collected in rocky bottoms with thalli growing on dead coral. This difference was attributed to changes in concentrations of the meroditerpene atomaric acid (**1**). Meanwhile, the major metabolite found in *C. crispatus* samples was dictyol B acetate (**2**). **Conclusions:** NMR metabolomics of San Andrés brown algae differentiated species based on lipid content and metabolic complexity. Notably, prenylated-guaiane diterpenes characterized *C. crispatus*, and meroditerpenoid concentrations varied in *S. zonale*. Temporal lipid variations were observed in *P. gymnospora*, while juvenile *Dictyota* spp. presented a less complex metabolic signature.

## 1. Introduction

Brown algae constitute a diverse group of photosynthetic organisms found mostly in marine and in some freshwater environments, playing critical ecological roles in numerous ecosystems [1]. Many species from the order Dictyotales are significant components of tropical marine ecosystems, providing food sources [2], habitats [3] and having a significant role in processes such as nutrient cycling [4] and atmospheric carbon fixation [5]. As a consequence of several environmental and anthropogenic factors, stony coral populations throughout the Caribbean and the Western Atlantic have declined [6], leading to the proliferation of opportunistic benthic organisms such as sponges, cyanobacteria and brown algae, particularly those from the genus *Dictyota*, which have emerged as a dominant group in these environments. This group has caused negative effects in reef ecosystems, leading to a generalized loss of biodiversity, hence affecting local fisheries, tourism activities, loss of coastal protection and endangering food security for local communities [7].

Algae of the Order Dictyotales produce a wide array of compounds, with different types of biological activities, including antifungal [8], antibacterial [9,10], antiviral [11,12] and cytotoxic effects [13]. Additionally, some of those compounds act as potent deterrents against herbivory [14] and fouling [15]. Several species in this order (genera *Dictyota*, *Stypopodium* and *Canistrocarpus*) have undergone extensive research due to their metabolic diversity, particularly diterpenoids [16,17]. However, it has been documented those algae collected from different locations differ in their metabolite production depending on environmental factors and time of collection, hence it is important to characterize compounds ascribed to particular species even if they have been previously studied [16]. All this preliminary information could be used for speed-up studies that intend to characterize previously studied species in order to avoid re-isolation of well-known compounds and then focus on those compounds that are differentially expressed depending on environmental factors [17]. In this context, metabolomics is a reliable approach for the chemical study of those samples [18,19].

Compared to terrestrial biota (mostly terrestrial plants), to date, there are relatively few metabolomic studies on marine organisms. Metabolomic profiling approaches have facilitated the establishment of interspecific phylogenetic relationships previously unresolved through morphological and molecular approaches in sponges [20], zoanthids [21], dinoflagellates [22] and soft corals [23]. There are few metabolomic studies in marine algae, although due to their metabolic diversity, rapid growth and nutrient assimilation capacity, they are promising model organisms to apply these tools.

The metabolic diversity in samples from four species of brown algae of the genus *Lobophora*, collected in New Caledonia, was evaluated by three analytical platforms: High-Performance Liquid Chromatography–Mass Spectrometry (HPLC–MS), Gas Chromatography-Mass Spectrometry (GC–MS) and Nuclear Magnetic Resonance Spectroscopy (NMR). The best technique for the taxonomic discrimination within the species *Lobophora monticola*, *L. obscura*, *L. rosacea* and *L. sonderii* was HPLC–MS, establishing the compounds known as lobophorenols as markers [18]. Additionally, a spatial and temporal comparison of the four species showed changes in the samples metabolic profile related to the sampling time (13 months) and sampling area [19]. In another study carried out on Egyptian coast with the green alga *Ulva fasciata*, the red alga *Pterocladia capillacea*, the brown alga *Sargassum hornschuchii* and the cyanobacterium *Arthrospira platensis*, using HPLC–MS, it was possible to correlate their metabolic expression with their cytotoxic potential against two cell lines, finding that extracts from *A. platensis* were active against the PC3 and MDA-MB-231 cancer cells, and identifying the compounds campestanol, glutamylglycine and rosmanol as being responsible for the observed activity [24].

There have been few studies on the chemistry and metabolic profiling of brown algae in Colombia, and specifically, samples from the island of San Andres have not been analyzed yet. Thus, the main objective of this research was to characterize the metabolic profiles of the most abundant brown algae in the shallow marine habitats of the northern area of the island, by means of non-targeted metabolomic analyses using proton nuclear magnetic resonance (^1^H-NMR) spectroscopy, considering the taxonomic identity, collection date and growth substrate as the variables that could contribute to differentiate among the sampled species.

## 2. Materials and Methods

### 2.1. Collection of Biological Material

The biological material was collected on the island of San Andres, by free diving in coralline patches and sandy bottoms at a depth range between 0 to 10 m. Algal samples were cleaned in situ, removing epiphytic organisms and excess sand and immediately stored in resealable bags and frozen at −20 °C. Samples were transported to the Laboratories of Universidad Nacional de Colombia at Bogotá for subsequent analysis. Samples were collected under the collecting permit granted by the Ministerio de Ambiente y Desarrollo Sostenible (Contrato Marco de Acceso a Recursos Genéticos y sus productos derivados No. 121, otrosí No. 7). Samples were identified by Brigitte Gavio Ph.D. and N. Rincón-Díaz M.Sc. based on morphological parameters. Vouchers of samples were deposited in the collection of Herbario de la Pontificia Universidad Javeriana, under the codes HPUJ30925, HPUJ30926, HPUJ30927 and HPUJ30928.

### 2.2. General Experimental Procedures

The organic solvents dichloromethane (DCM), hexane (Hex), ethyl acetate (EtOAc) and methanol (MeOH), analytical reagent grade (Merck, Darmstadt, Germany), were used for the extraction process, thin-layer chromatography (TLC) and flash column chromatography (CC). Column chromatography was performed using Silica Gel 60 (70–230 MESH ASTM, Merck, Darmstadt, Germany) as stationary phase, and mixtures of AR-quality solvents (Merck, Darmstadt, Germany) were used as mobile phases (Hex/EtOAc/MeOH).

Nuclear Magnetic Resonance spectra were recorded on an Advance Neo 400 MHz (Brucker, Billerica, MA, USA), equipped with a BBO probe and using 99.8% deuterated chloroform (Merck, Darmstadt, Germany). GC–MS analyses were performed on a GC7890 gas chromatograph (Agilent Technologies, Santa Clara, CA, USA) coupled with an MSD-5975C Mass Spectrometer (Agilent Technologies, Santa Clara, CA, USA) at 70 eV electron impact (IE) ionization mode. An HP-5MS (5% phenylmethyl-siloxane) column of 60 m × 250 μm × 0.25 μm was employed, and the temperatures of the injection port, ionization chamber and transfer line were set at 270, 290 and 290 °C, respectively. The GC oven temperature was programmed from 40 °C (1 min) to 100 °C at 60 °C/min, then to 260 °C, at 4 °C/min and finally to 290 °C (15 min) at 10 °C/min. Helium (99.99%) was used as the carrier gas at a flow rate of 1 mL/min. Under the same chromatographic conditions, a mixture of standard paraffins (C14–C26) were injected to calculate the Retention Index (RI) according to the Kovats method. Fraction analyses were performed on a HPLC Dionex UltiMate 3000 device (Thermo Fisher Scientific, Waltham, MA, USA) coupled sequentially with a DAD detector (Thermo Fisher Scientific, Waltham, MA, USA) and 85L ELSD detector (Sedere, Olivet, France) operated with a gain of 10 arbitrary units. Preparative HPLC separations were performed on HITACHI 6000 A equipment (Hitachi, Chiyoda, Tokyo) with a UV/Vis detector L-4250 (Hitachi, Chiyoda, Tokyo). For HPLC procedures, acetonitrile Lichrosolv^®^ from Merck (Merck, Darmstadt, Germany) and ultrapure water Type I Milli-Q Merck Millipore (Merck, Darmstadt, Germany) filtered through a 2.2 μm membrane.

### 2.3. Comparison of the Metabolic Profile Between Species and Non-Targeted Metabolomic Experiment

For the characterization of the metabolic profiles of the collected algae, a non-targeted experiment was designed using ^1^H-NMR spectroscopy. Once the biological material was collected and preliminarily identified, the samples were separated by removing sand and epiphytic organisms to immediately be frozen at −20 °C and kept in optimal conditions for transport. To carry out metabolic profiling, 1 g of fresh biological material was used, which was extracted with a dichloromethane/methanol mixture (1:1) for 30 min in an ultrasound bath (procedure performed in triplicate). The extracts were filtered and dried under reduced pressure (water bath temperature 20 °C), then suspended in water to perform a 1:1 water/dichloromethane partition; the aqueous phases were discarded (mainly salts), then the organic fractions were dried under reduced pressure and stored at −20 °C for the metabolomic experiment.

Spectra were obtained from 5 mg of crude extract dissolved in 500 μL of CDCl_3_ with the following experimental conditions: zg30 pulse sequence, room temperature 25 °C, 32 scans, window size from −2 to 12 ppm, FID size 64K, pulse width PW = 30, relaxation time = 2.0 s, dummy scans: 2.0 s, receiver gain: 101. For processing, each spectrum was adjusted using the MestreNova 10.1 software by adjusting the phase and baseline manually. Additionally, spectra were calibrated with the residual signal of CDCl_3_ at δ 7.26 ppm. Finally, “binning” was executed in the same software, creating the variables from 0 to 9 ppm in intervals of 0.04 ppm, thus generating a data matrix for multivariate analyses. In total, 88 ^1^H-NMR spectra were obtained.

Data analysis was performed using the online tool MetaboAnalyst 6.0 “https://www.metaboanalyst.ca/ (accessed on 18 January 2025)”, specifically the Statistical Analysis (one factor) module. In the first step, no need for data filtering from the software was required. Data normalization was performed by the sum tool and were then adjusted to a square root model in order to reduce skewness and stabilize variance; finally, they were scaled by the Pareto method, which balances the importance of the variables and enhances the identification of important metabolites. With the processed data, the following chemometric analyzes were carried out: principal component analysis (PCA), hierarchical cluster analysis (HCA) and partial least-squares discriminant analysis (PLS-DA), which, in addition to presenting the Scoreplot, made it possible to obtain the important variables in projection (VIP), which permitted the identification of which signals (chemical shifts) were responsible for the formation of the observed groups.

### 2.4. Extraction and Isolation Elucidation of Compounds (**1**) and (**2**)

Atomaric acid (**1**) was isolated from *Stypopodium zonale* samples. Starting with 200 g of frozen algae, extraction was performed using a dichloromethane/methanol mixture (1:1, DCM/MeOH) in an ultrasonic bath with chilled water (300 mL, 10 °C) for 30 min. The resulting extract was dried under vacuum at 40 °C. Subsequent partitioning with DCM and water (1:1) yielded 6.7 g of a tar-like black organic extract. Then, 100 mg aliquot of this extract was then subjected to preparative HPLC on a reversed-phase RP-18 column (Kromasil 100-5, 10 × 250 mm, 5 μm). Elution was achieved using a continuous acetonitrile/water (ACN/H_2_O) gradient: 10:90 (*v*/*v*) for the first minute, increasing to 50:50 (*v*/*v*) by minute 5, followed by a second gradient to 80:20 (*v*/*v*) by minute 15 and finally reaching 100% ACN from minute 20 to minute 45. Fraction F3 (18 mg), identified as atomaric acid (**1**), was confirmed by comparison of its experimental ^1^H and ^13^C NMR spectroscopic data with those reported by Dorta et al. (2003) [25].

To isolate the most abundant metabolites from *C. crispatus* samples, 430 g of frozen algae were subjected to exhaustive extraction using a mixture of dichloromethane and methanol (in a 1:1 ratio DCM/MeOH) in an ultrasonic bath with cooled water (300 mL, 10 °C, for 30 min). Subsequently, the extract was dried under vacuum at 40 °C. The crude extract was then partitioned using a mixture of DCM and water (1:1), resulting in 8.9 g of a dark greenish, sticky organic extract. The organic fraction underwent separation via normal-phase flash chromatography (10 mL/min flow rate) in silica gel 60 (70–230 MESH ASTM, Merck) using a discontinuous gradient of increasing polarity (hexane, ethyl acetate and methanol), yielding 12 fractions (F1–F12). Fractions F2 (32 mg) and F3 (49 mg) were further separated using the same preparative HPLC methodology utilized to isolate (**1**). This separation process yielded compounds (**2**) dictyol B acetate (17 mg) and pachydictyol A (8 mg). The identities of the isolated compounds were verified by comparison of their ^1^H and ^13^C-NMR spectra (Figures S7 and S9) to those reported by Caamal-Fuentes et al. (2014) [26], and by GC–MS analyses to those reported by Freitas et al. (2007) [27] and Pinheiro et al. (2019) [28].

Atomaric acid (**1**): ^1^H NMR (400 MHz, CDCl_3_) 6.69 (1H, d, j = 3.0, 6′-H), 6.54 (1H, d, J = 2.6, H-4′), 3,73 (3H, s, COCH_3_), 2.22 (3H, s,H-7′), 2.33 (1H, m, H-11), 2.29 (1H, m, H-13), 2.37 and 1.95 (2H, m, H-9), 2.86 and 2.24 (2H, d, J = 14, H-1), 1.82 (1H, m, H-4), 1.81 and 1.61 (2H, m, H-12), 1.73 and 1.52 (2H, m, H-8), 1.73 and 1.21 (2H, m, H-3), 1.51(1H, m, H-5), 1,68 (3H, m, H-19), 1.69 (3H, s, H-20), 1.38 (1H, m, H-7), 1.16 (3H, d, J = 6.9, H-15), 1.03 (3H, s, H-17), 0.93 (3H, s, H-16). ^13^C-NMR (100 MHz, CDCl_3_): 180.4 (C-14), 152.5 (C-5′), 149.2 (C-2′), 132.9 (C-10), 127.1 (C-1′), 124.1 (C-3′), 123.4 (C-18), 114.5 (C-6′), 113.6 (C-4′), 55.5 (COCH3), 53.2 (C-11), 42.0 (C-7), 40.9 (C-2), 38.9 (C-6), 36.6 (C-6), 35.4 (C-3), 35.4 (C-1), 33.0 (C-13), 25.5 (C-4), 25.1 (C-12), 23.5 (C-9), 22.4 (C-8), 20.8 (C-16), 20.4 (C-19), 20.4 (C-20), 17.9 (C-17), 16.5 (C-7′).

Dictyol B acetate (**2**): GC–MS m/z (%): m/z 346 (0) [M]+ and the ions at m/z 286 (14); 268 (18); 225 (12); 197 (25), 186 (25), 173(17), 157 (100); 105 (38); 69 (71); 55 (36); 43 (57), 41 (58). ^1^H NMR (400 MHz, CDCl_3_) 5.33 (1H, m, H-3), 5.17 (1H, m, H-9), 5.14 (1H, m, H-14), 4.97 and 4.92 (2H, brs, H-18), 3.91 (1H, dd, J = 8.2 and 3.7, H-6), 2.14 (3H, s, COOCH_3_), 2.60 and 2.26 (2H, m, H-2), 1.54 and 1.26 (2H, m, H-12), 2.07 and 1.95 (2H, m, H-13), 1.82 and 1,67 (2H, m, H-8), 1.79 (3H, brs, H-17), 1.68 (3H, s, H-16), 1.60 (3H, s, H-20), 1.68 (1H, m, H-7), 1.61 (1H, m, H-11) 1.01 (3H, d, J = 6.1, H-19). ^13^C-NMR (100 MHz, CDCl_3_): 149.6 (C-10), 140.9 (C-4), 131.9 (C-15), 124.6 (C-14), 124.0 (C-3), 104.8 (C-18), 77.4 (C-9), 74.6 (C-6), 61.1 (C-5), 44.0 (C-7), 43.1 (C-1), 35.0 (C-12), 34.8 (C-11), 33.8 (C-2), 30.3 (C-8), 25.9 (C-16), 25.7 (C-13), 17.8 (C-20), 17.5 (C-19), 15.8 (C-17).

Pachydictyol A: GC–MS m/z (%): m/z 288 (14) [M]+ and the ions at m/z 270 (16), 255 (4), 227 (4), 213 (8), 203 (33), 199 (16), 188 (9), 175 (19), 173 (14), 159 (89), 157 (28), 145 (32), 131 (36), 120 (68), 107 (83), 105 (89), 91 (67), 82 (85), 69 (97), 55 (57), 41 (100). ^1^H NMR (400 MHz, CDCl_3_) 5.33 (1H, m, H-3), 5.13 (1H, m, H-14), 4.73 (2H, brs, H-18), 3.92 (1H, d, J = 7.8, H-6), 2.49 and 2.21 (2H, m, H-2), 2.62 and 2.10 (2H, m, H-9), 2.24 and 1.53 (2H, m, H-12), 2.04 and 1.95 (2H, m, H-13), 1.80 (3H, brs, H-17), 1.68 (3H, s, H-16), 1.61 (3H, s, H-20), 1.55 (1H, m, H-7), 1.50 (2H, m, H-8), 1.20 (1H, m, H-11), 0.99 (3H, d, J = 6.0, H-19). ^13^C NMR (100 MHz, CDCl_3_): 152.7 (C-10), 141.5 (C-4), 131.7 (C-15), 124.8 (C-14), 124.1 (C-3), 107.2 (C-18), 40.7 (C-9), 75.2 (C-6), 60.4 (C-5), 47.9 (C-7), 46.2 (C-1), 35.1 (C-12), 34.8 (C-11), 33.9 (C-2), 23.6 (C-8), 25.8 (C-16), 25.7 (C-13), 17.8 (C-20), 17.6 (C-19), 16.0 (C-17).

## 3. Results

### 3.1. Metabolic Profiling of Brown Algae Samples of the Order Dictyotales

San Andres island is located in the southwestern Caribbean along the Nicaraguan rise. The island’s coral reefs, seagrass beds and mangroves support a rich diversity of marine life, including numerous species of fishes, crustaceans and mollusks [29]. These ecosystems are essential for the breeding and feeding of many marine organisms. The sampled site, located at the northern tip of the island between depths of 0 and 10 m, consisted of three marine shallow habitats: sandy, rocky bottoms and dead coral. The site was selected due to the apparent low influence from human activities and relative high diversity of benthic brown algae. This location experiences a strong influence from winds and currents and has some small coral patches of *Acropora palmata*, *Porites* sp., *Pseudodiploria strigosa* and *P. clivosa*. Four collections were carried out at the site between 2018 and 2019, revealing that brown algae diversity (species richness) was variable across the two years of sampling (Table 1).

During the first collection in May 2018, only two species of brown algae (*Dictyopteris delicatula* and *Stypopodium zonale*) were found. However, in the collections from January and May 2019, *Canistrocarpus crispatus*, *Padina gymnospora*, *D. delicatula* and *S. zonale* were identified as the most abundant brown algae in the area. In October 2019, juvenile thalli of *Dictyota* spp. were the only category, but their size was so small (>1 cm) that it was not possible to taxonomically identify those algae to the species level (see Table S1 for the complete samples list).

**Figure 1:**
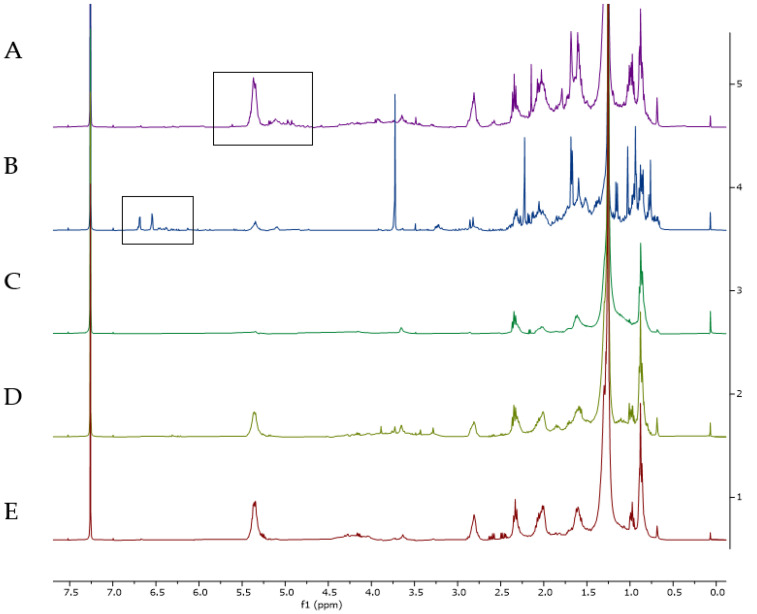
Overlapping of ^1^H-NMR (CDCl_3_, 400 MHz) spectra from organic extracts from the different species of brown algae collected in this study. (**A**). *Canistrocarpus crispatus*, (**B**). *Stypopodium zonale*, (**C**). juvenile thalli of *Dictyota* spp., (**D**). *Dictyopteris delicatula*, (**E**). *Padina gymnospora*. Regions of highlighted signals are shown by a black rectangle.

When comparing the ^1^H NMR spectra from the organic extracts of each of the collected species (Figure 1), the most intense signals corresponded to lipids such as fatty acids, compounds characterize by the intense peak at δ 1.26. Meanwhile, the spectra of *C. crispatus* stood out for having greater complexity of signals in the olefinic range (δ 4.5–5.5; see Figure 1 spectrum A), and those from *S. zonale* showed signals between δ 6.0 and 6.7 ppm corresponding to aromatic protons in the oxygenated rings (Figure 1 spectrum B).

### 3.2. ^1^H-NMR Species Comparison Metabolomic Experiment

**Figure 2:**
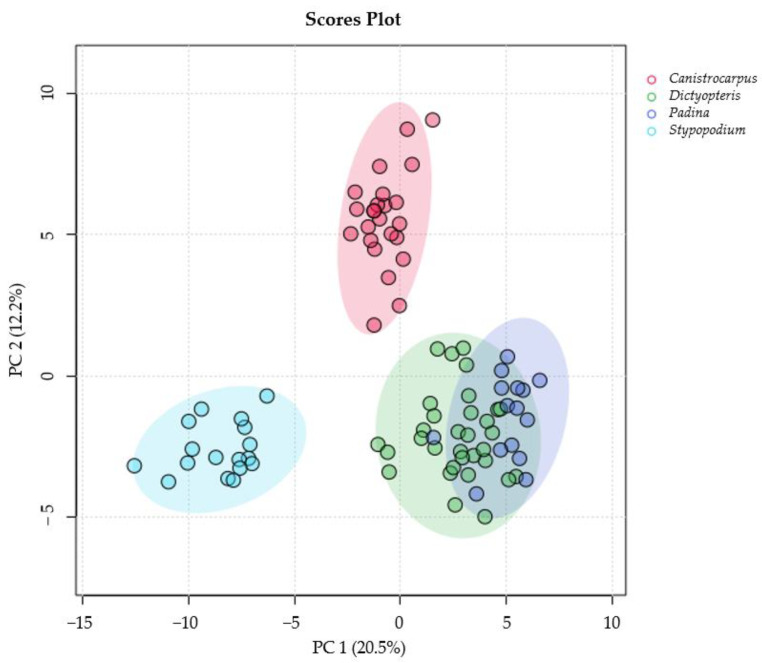
Principal component analysis scores plot (n = 88) for organic extracts from *Canistrocarpus crispatus* (red), *Stypopodium zonale* (light blue), *Dictyopteris delicatula* (green) and *Padina gymnospora* (blue) using component 1 (20.5%) and component 2 (12.2) in the X and Y axes, respectively.

To compare the metabolic production among the four species of brown algae considered in this study (*C. crispatus*, *D. delicatula*, *P. gymnospora* and *S. zonale*), a non-targeted metabolic profiling experiment was conducted. Eighty-eight algal samples were extracted using a 1:1 mixture of dichloromethane and methanol. The extracts were then partitioned with a 1:1 mixture of water and dichloromethane. Subsequently, the ^1^H-NMR spectra of the organic extracts were acquired and processed. A multivariate matrix was created using these data, generating bins from 0–9 ppm with intervals of 0.04 ppm (resulting in 88 samples × 221 variables). This matrix was used to perform exploratory multivariate analyses, including principal component analysis (PCA), hierarchical cluster analysis (HCA) and partial least-squares discriminant analysis (PLS-DA). The goal was to determine the metabolic differences between species. The PCA (Figure 2 and Figure S1) and HCA (Figure S2) showed that there were differences between the NMR data from the organic extracts of the four species; samples from *C. crispatus* and *S. zonale* clustered apart from other species, whereas those from *P. gymnospora* and *D. delicatula* showed minor differences among them.

**Figure 3:**
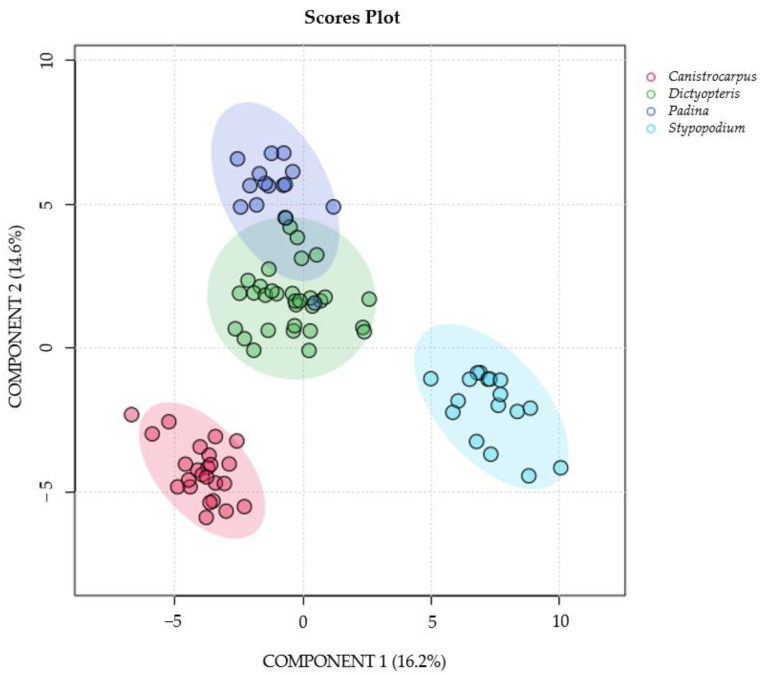
Partial least discriminant analysis PLS-DA scores plots (n = 88) for organic extracts from *Canistrocarpus crispatus* (red), *Stypopodium zonale* (light blue), *Dictyopteris delicatula* (green) and *Padina gymnospora* (blue) using component 1 (16.2%) and component 2 (14.6%) in the X and Y axes. respectively. Cross validation R^2^: 0.87%, Q^2^: 0.81%.

The scores plot of the PLS-DA using the species identification as supervising variables (Figure 3 and Figure S3) with component 1 (16.2%) and component 2 (14.6%) in the X and Y axes, respectively (R^2^: 0.87%, Q^2^: 0.81% see Table S2), allowed the visualization of distinct groups based on algal species. On one hand, species with reported diterpenoids such as *C. crispatus* and *S. zonale* clustered independently from the other samples. On the other hand, the NMR spectra of *P. gymnospora* and *D. delicatula* were closer to each other in a two-dimensional space, indicating their metabolic resemblance, mainly dominated by the signals of fatty acids as shown in Figure 1, spectrum D and spectrum E.

**Figure 4:**
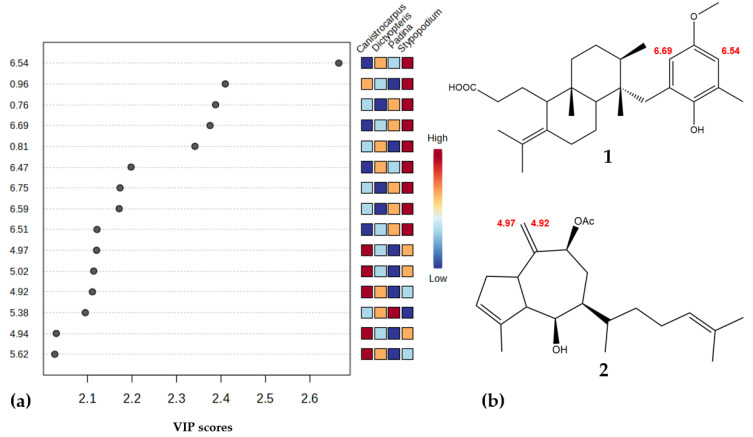
(**a**): VIP plot of the PLS-DA which discriminates the most important variables in the formation of groups observed in the S-plot for the organic fractions of each algal species. The red color indicates that the signals are of greater intensity; in this case, the signals at δ 6.54, 0.96, 0.76, 6.69, 0.81, 6.47, 6.75, 6.59 and 6.51 were assigned to *S. zonale*; the signals at δ 4.97, 5.02, 4.92 and 4.94 were assigned to *C. crispatus;* and signal δ 5.38 was assigned to *P. gymnospora*. (**b**): Structures of the chemical markers isolated from *S. zonale* (atomaric acid (**1**)) and *C. crispatus* (dictyol b acetate (**2**)). Chemical shifts identified as VIPs are highlighted in red.

The variables important in projection (VIPs), in this case, chemical shifts in the ^1^H-NMR spectra (Figure 4a) from the PLS-DA analysis, indicate signals corresponding to chemical biomarkers, specifically meroditerpenes for *S. zonale* and diterpenoids for *C. crispatus*. Chemical shifts in the range from δ 6.50 to 6.74 are characteristic for protons in the aromatic ring substructures of meroditerpenes commonly found in *S. zonale*, while those between δ 4.92 and 5.02 correspond to protons on double bonds found in the carbon skeleton structures of diterpenoids in *C. crispatus*. Upon isolating the most abundant specialized metabolites (vide supra), it was observed that the signals identified in the multivariate analysis (PLS-DA) corresponded to substructures found in terpenoids reported for each species. Specific chemical markers identified include signals at δ 6.54 and 6.69 for *S. zonale* (Figure 4b, Figures S4 and S5 and Table S3), which are characteristic of the two aromatic protons of atomaric acid (**1**) [25]. For *C. crispatus* (Figure 4b, Figures S6 and S7 and Table S3), signals at δ 4.92 and 4.97 correspond to protons of a terminal vinyl group reported for compound (**2**) dictyol B acetate [26]. Additionally, pachydictyol A (Figures S8 and S9 and Table S3) was isolated and identified from the *C. crispatus* samples by 1D- and 2D-NMR, although none of its ^1^H-NMR signals were detected in the metabolomic experiment. Both dictyol B acetate and pachydictyol A were identified by GC-MS (see Figures S10–S13).

### 3.3. Effect of Collection Date and Growth Substrate on the Metabolic Profiles of Brown Algae Species

To investigate the potential impact of collection dates (May 2018, January 2019 and May 2019) and substrate types (dead coral, coralline rock or sand) on the metabolic production in the collected brown algae, a metabolic profiling experiment was conducted. The experiment followed the same specifications used for interspecific comparison, incorporating as supervising variables, the collection date and growth substrate.

**Figure 5:**
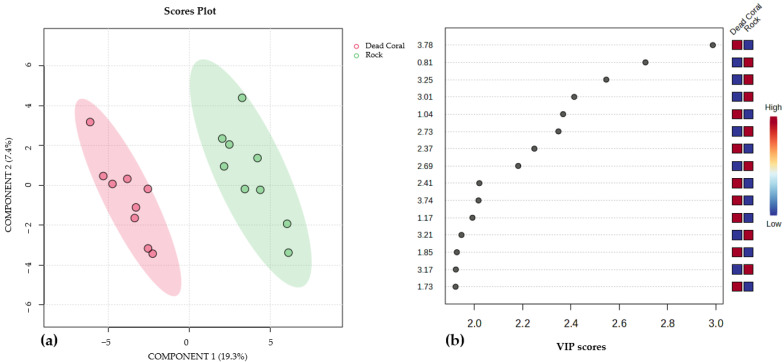
(**a**): Partial least discriminant analysis PLS-DA scores plots (n = 18) for *S. zonale* samples growing on dead coral (red) and coralline rock (green) substrates using component 1 (19.3%) and component 2 (7.4%) in the X and Y axes, respectively. Cross validation R^2^: 0.96%, Q^2^: 0.66% (see also Table S4). (**b**): VIP plot of the PLS-DA which discriminates the most important variables in the formation of the groups observed in the S-plot for each type of substrate in the organic fractions of *S. zonale*. The red color indicates that the signals are of greater intensity; in this case, the signals at δ 3.78, 1.04, 2.37, 2.41, 3.74, 1.17, 1.85 and 1.73 were assigned to the samples collected on dead coral, and those at δ 0.81, 3.25, 3.01, 2.73, 2.69, 3.21 and 3.17 were assigned to the samples collected on coralline rock.

The only species in which the growth substrate had a discernible effect on metabolic profile was *S. zonale* (see Figure 5a and Figure S14). Samples collected on dead coral exhibited a higher concentration of atomaric acid (**1**) compared to thalli collected on coralline rock. This can be observed in the relative intensity of the methoxy group peak at δ 3.78 ppm, contrasting with the intense peak at δ 1.26 ppm corresponding to the signals for the aliphatic methylene (CH_2_)-chains (see Figure 5b and Figure S15).

**Figure 6:**
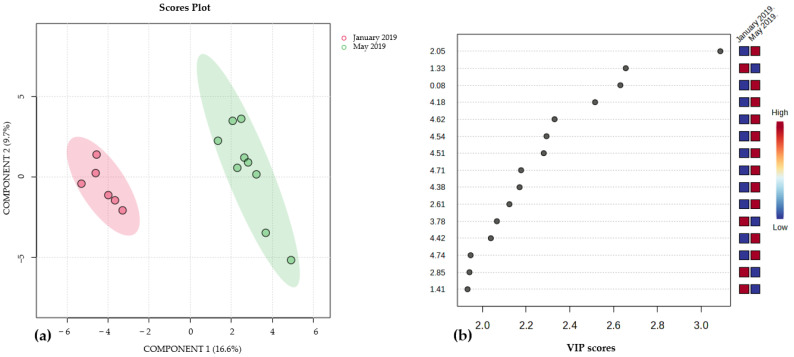
(**a**): Partial least discriminant analysis PLS-DA scores plots (n = 15) for *P. gymnospora* samples comparing collection dates, January 2019 and May 2019, using component 1 (16.6%) and component 2 (9.7%) in the X and Y axes, respectively. Cross validation R^2^: 0.98%, Q^2^: 0.76% (see also Table S5). (**b**): VIP plot of the PLS-DA which discriminates the most important variables in the formation of the groups observed in the S-plot for collection date in samples of *P. gymnospora*. The red color indicates that the signals are of greater intensity; in this case, the signals at δ 1.33, 3.78, 2.85 and 1.41 were assigned to the samples collected in January 2019, and the signals at δ 2.05, 0.08, 4.18, 4.62, 4.54, 4.51, 4.71, 4.38, 2.61, 4.42 and 4.44 were assigned to samples collected in May 2019.

On the other hand, it was found that only for *P. gymnospora*, the collection date had an impact on the metabolic production. Samples collected in January 2019 and those collected in May 2019 exhibited a distinct separation in the multivariate analysis (Figure 6a and Figure S16). The signals responsible for separating these groups, located between 3.0 and 4.5 ppm (see Figure 6b and Figure S17), are indicative of molecules containing oxygenated protons, such as glycolipids; however, those compounds were not isolated in this study.

### 3.4. Juvenile Dictyota spp. Metabolic Profile

**Figure 7:**
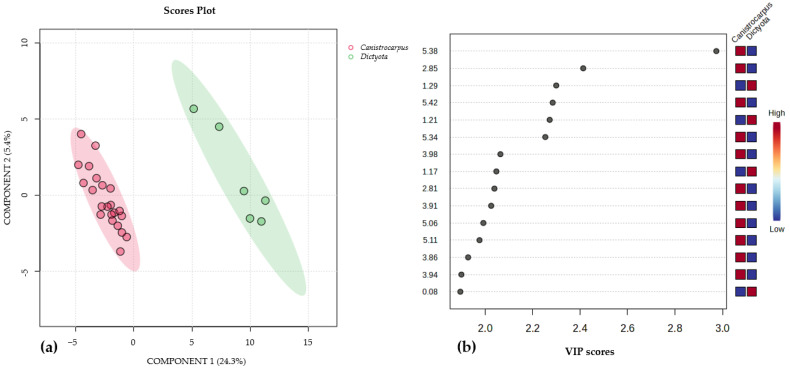
(**a**). Partial least discriminant analysis PLS-DA scores plots (n = 28) comparing young thalli of *Dictyota* spp. and *C. crispatus* samples using component 1 (24.3%) and component 2 (5.4%) in the X and Y axes, respectively. Cross validation R^2^: 0.98%, Q^2^: 0.90% see also Table S6. (**b**). VIP plot of the PLS-DA which discriminates the most important variables in the formation of the groups observed comparing young thalli of *Dictyota* spp. and *C. crispatus* samples. The red color indicates that the signals are of greater intensity; in this case, the signals at δ 5.38, 2.85, 5.42, 5.34, 3.98, 2.81, 3.91, 5.06, 5.11, 3.86 and 3.94 were assigned to *C. crispatus,* whereas the signals at δ 1.29, 1.21, 1.17 and 0.08 were assigned to juvenile *Dictyota* spp.

Finally, a comparison of metabolic production was conducted between samples of juvenile thalli of *Dictyota* spp. collected in October 2019 and populations of *C. crispatus* (collected in January and May 2019) due their morphological resemblance. In the multivariate analysis (see Figure 7a and Figure S18), a distinct separation was observed between the analyzed extracts, as the extracts from *Dictyota* spp. exhibited few signals, mainly corresponding to saturated fatty acids (Figure 1, spectrum C). Conversely, extracts of *C. crispatus* samples clearly displayed signals indicative of compounds with double bonds in their structure, such as terpenoids and unsaturated fatty acids (Figure 7b and Figure S19). However, further identification of minor compounds was not accomplished due to low intensity of signals in the spectrum.

## 4. Discussion

The diversity of brown algae in the collection site was found to be highly variable over time. This variability can be attributed to their rapid growth rate and opportunistic life history, as well as their chemical defenses, which help prevent consumption by grazing organisms such as fish and certain invertebrates such as sea urchins and mollusks [30]. Information on the lifespan of individual brown algae species from the order Dictyotales may not be readily available. Marine algae thrive in a wide range of habitats, from intertidal zones to deeper waters, and their lifespan can be influenced by factors such as water temperature, nutrient availability and grazing pressure [31].

^1^H-NMR spectroscopy allowed us to detect the class and relative quantity of metabolites produced in the collected algae. This approach enabled the identification of the most abundant specialized metabolites synthesized by different species of brown algae in the order Dictyotales. These metabolites were identified from crude extracts that are low-cost and easy to prepare. Additionally, the ^1^H-NMR technique provides metabolic profiles of each sample with high reproducibility in a short processing time. The use of this technique adds significant value to the results of metabolomic experiments, as it allows for efficient analysis and interpretation of large amounts of spectroscopic data, detecting variability in metabolic production between samples.

The metabolic profiles of the brown algae in this study are comparable to similar reports in which lipidic compounds were identified as the major metabolites in species in the order Dictyotales [30], among which polyunsaturated fatty acids [32], fucosterol [33] and oxylipins [34] stand out. Both multivariate analyses were performed, the PCA and the PLS-DA using ^1^H-NMR spectra explained percentages of variability in the samples similar to those obtained in a comparison of various species of the genus *Lobophora* on the island of New Caledonia in a study with similar type of data [18]. The juvenile *Dictyota* spp. samples analyzed from October 2019 exhibited fewer signals in their ^1^H-NMR spectra compared to *C. crispatus*, evidencing poor metabolic production. In a feeding preference experiment using *Arbacia punctulata* and *Dictyota ciliolata*, the sea urchin preferentially consumed the young apices of the alga compared to older plant tissue; this preference was due by the fact that diterpene production in the genera *Dictyota* is associated with increased growth and size of the thallus [35].

The metabolic profiling study allowed us to detect those thalli of *Stypopodium zonale* collected at San Andrés Island produce atomaric acid (**1**) as their major specialized metabolite, just like populations of *S. zonale* from Belize and the Florida Keys [36]; Tenerife, Canary Islands [25]; Buzios, Brazil [37]; and the Abrolhos archipelago, Brazil [38]. This compound, together with other meroditerpenes, prevents the algae from being consumed by fish and herbivorous invertebrates, thus giving it the ability to survive in a large variety of marine environments [39]. The difference in the concentration of methoxylated compounds, such as the atomaric acid between the two growth substrates, may be explained since the algae were colonizing dead corals and may have required a greater deterrent capacity against herbivorous invertebrates. In a feeding deterrent experiment, it was found that pure atomaric acid (**1**), compared to other meroditerpenes, was highly effective against grazing activities by the crab *Pachygrapsus transversus* and the sea urchin *Lytechinus variegatus* [37].

Regarding *Canistrocarpus crispatus*, only one study so far has addressed the metabolic production of this algae. In the coasts of Brazil, three diterpenes of the dolastane type were isolated from this species [40]. Those data differ from the results obtained in this research, where the detected diterpenes dictyol B acetate (**2**) and pachydictyol A belonged to the prenylated-guaiane type. In this study, the diterpene produced in the greatest concentration was dictyol B acetate (**2**), a compound commonly produced by several species in the genus *Dictyota*, such as *D. dichotoma var implexa* [41], *D. caribaea* [42], *D. mertensii* [27,28] and *D. ciliolata* [26]. Dictyol B acetate has several properties that could contribute to the algal chemical defenses [43]. For example, its antimicrobial properties could protect algae from bacterial or fungal infestation [44]. The molecule dictyol B acetate has low palatability, protecting the algae against herbivory by grazing fish and invertebrates [35,45]. Additionally, its allelopathic effects could inhibit the growth or development of neighboring species, thus providing the algae with a competitive advantage in their habitats [46].

## 5. Conclusions

The multivariate analyses conducted on ^1^H-NMR spectra of crude extracts from brown algae collected at various times enabled the categorization and classification of samples from shallow environments from San Andrés Island in two groups: extracts rich in lipid compounds (*D. delicatula* and *P. gymnospora*) and metabolically complex extracts (*C. crispatus* and *S. zonale*). These results allowed the identification of major specialized metabolites based on the signals detected in the multivariate analysis. The samples of *C. crispatus* showed that the studied populations of this species produced prenylated-guaiane diterpenes (dictyol B acetate (**2**) and pachydictyol A) as their major specialized metabolites. Differences in the metabolic profiles were observed in samples of *S. zonale* collected on dead coral versus coralline rock, which were attributed to variations in the concentration of atomaric acid (**1**). Similarly, disparities were detected in the metabolic profiles of *P. gymnospora* collected in January 2019 and May 2019, likely explained by the changes in lipid production. The relative simplicity of the metabolic profile observed in juvenile *Dictyota* spp., the dominant benthic species in October 2019, contrasted sharply with the metabolic complexity of other brown algae species in the northern San Andres Island marine ecosystems.

## Figures and Tables

**Table 1:** Species of brown algae collected in San Andres Island, including the date of collection and growth substrate.

Species	Collection Months	Substrate(s)
*Canistrocarpus crispatus*	January 2019, May 2019	Coralline rock, Dead Coral
*Stypopodium zonale*	May 2018, January 2019, May 2019	Coralline rock, Dead Coral
*Padina gymnospora*	January 2019, May 2019	Coralline rock, Sand
*Dictyopteris delicatula*	May 2018, January 2019, May 2019	Coralline rock, Dead Coral
*Dictyota* spp.	October 2019	Coralline rock

## Data Availability

The raw data of this article can be found online on https://figshare.com/articles/dataset/de_la_Roche_et_al_DATA_zip/28888286?file=54029924 (accessed on 18 January 2025) on free access.

## Supplementary Materials

The following supporting information can be downloaded at: https://www.mdpi.com/article/10.3390/metabo15050305/s1, Table S1. List of the species used for the metabolomic analysis; organic extracts were made with individual thali of each brown algae. Table S2. Cross validation of the model generated in the PLS-DA of the ^1^H-NMR spectra of organic fractions of brown algae collected in shallow environments north of the island of San Andrés including the R^2^ and Q^2^ statistics for components 1, 2 and 3. Table S3. ^1^H (CDCl_3_, 400 MHz) and ^13^C (CDCl_3_, 100 MHz) chemical shifts for compounds atomaric acid (1) isolated from *S. zonale*, and compounds dictyol B acetate (2) and pachydictyol A isolated from *C. crispatus*. Table S4. Cross validation of the model generated in the PLS-DA of the ^1^H-NMR spectra of organic fractions *S. zonale* collected in shallow environments (death coral and rock) north of the island of San Andrés including the R^2^ and Q^2^ statistics for components 1, 2, 3. Table S5. Cross validation of the model generated in the PLS-DA of the ^1^H-NMR spectra of organic fractions of *P. gymnospora* collected in shallow environments north of the island of San Andrés in different collection dates including the R^2^ and Q^2^ statistics for components 1, 2, 3. Table S6. Cross validation of the model generated in the PLS-DA of the ^1^H-NMR spectra of organic fractions of brown algae collected in shallow environments north of the island of San Andrés including the R^2^ and Q^2^ statistics for components 1, 2, 3. Figure S1. Principal component analysis scores plot (n = 88) for *Canistrocarpus crispatus* (red), *Stypopodium zonale* (light blue), *Dictyopteris delicatula* (green), *Padina gymnospora* (Dark Blue) using component 1 (20.5%), component 2 (12.2%) and component 3 (5.5%) in the axes. Figure S2. Hierarchical cluster analysis (HCA) dendrogram (n = 88) generated from ^1^H-NMR spectra of organic fractions of brown algae collected in shallow environments north of the island of San Andrés. *Canistrocarpus crispatus* (red), *Stypopodium zonale* (light blue), *Dictyopteris delicatula* (green), *Padina gymnospora* (Dark Blue) Pearson’s correlation was used as a distance measure and grouping was performed using the average algorithm. Figure S3. Partial least discriminant analysis PLS-DA scores plots (n = 88) for *Canistrocarpus crispatus* (red), *Stypopodium zonale* (light blue), *Dictyopteris delicatula* (green), *Padina gymnospora* (Dark Blue) using component 1 (16.2%), component 2 (14.6%) and component 3 (6.0%) in the axes. R^2^ 0.96% Q^2^: 0.87%. Figure S4. HPLC chromatogram of the organic extract from *Stypopodium zonale*, acquired using DAD (Pink 210 nm, Red 254 nm, Green 300 nm, Blue 366 nm) and ELSD (Black) detectors, shows an intense peak at 15 min corresponding to atomaric acid (1). Figure S5. ^1^H-NMR spectrum (CDCl_3_, 400 MHz) of atomaric acid (1) from *S. zonale*. Figure S6. HPLC chromatogram of the organic fraction F3 from *Canistrocarpus crispatus*, acquired using DAD (Pink 210 nm, Red 254 nm, Green 300 nm, Blue 366 nm) and ELSD (Black) detectors, shows a peak at 21 min corresponding to dictyol B acetate (2). Figure S7. ^1^H-NMR spectrum (CDCl_3_, 400 MHz) of dictyol B acetate (2) isolated from *C. crispatus*. Figure S8. HPLC chromatogram of the organic fraction F2 from *Canistrocarpus crispatus*, acquired using DAD (Pink 210 nm, Red 254 nm, Green 300 nm, Blue 366 nm) and ELSD (Black) detectors, shows a peak at 25 min corresponding to pachydictyol A. Figure S9. ^1^H-NMR spectrum (CDCl_3_, 400 MHz) of pachydictyol A isolated from *C. crispatus*. Figure S10. GC-MS chromatogram of the organic fraction F3 from *Canistrocarpus crispatus* shows a peak at 46 min identified as dictyol B acetate. Figure S11. Mass spectrum obtained by GC-MS (EI, 70 eV) of compound (2) dictyol B acetate. Figure S12. GC-MS chromatogram from the organic fraction F2 from *Canistrocarpus crispatus*, shows a peak at min 41 identified as pachydictyol A. Figure S13. Mass spectrum obtained by GC-MS (EI, 70 eV) of pachydictyol A. Figure S14. Principal component analysis PCA scores plot comparing brown algae species ^1^H organic extracts between growth substrate (Sand, dead coral and rock). A: *C. crispatus*, B: *D. delicatula*, C: *P. gymonspora*, D: *S. zonale*. Figure S15. Overlapping ^1^H-NMR spectra (CDCl_3_, 400 MHz) of organic extracts from *S. zonale* collected in dead coral (Blue) and rock (Red). Figure S16. Principal component analysis PCA scores plot comparing brown algae species ^1^H organic extracts between collection dates (May 2018, January 2019 and May 2019). A: *C. crispatus*, B: *D. delicatula*, C: *P. gymonspora*, D: *S. zonale*. Figure S17. Overlapping ^1^H-NMR spectra (CDCl_3_, 400 MHz) of organic extracts from spectra of *P. gymnospora* collected in January (Blue) and May 2019 (Red). Figure S18. Principal component analysis scores plots (n = 28) comparing juvenile *Dictyota* spp. and *C. crispatus* samples using component 1 (24.4%) and component 2 (9.4) in the axes. Figure S19. Overlapping ^1^H-NMR spectra (CDCl_3_, 400 MHz) of organic extracts from spectra of *C. crispatus* (Blue) and *Dictyota* spp. (Red).
