# Supplementary material for: Brown Algae from San Andres Island, Southwest Caribbean: A Nuclear Magnetic Resonance Spectroscopy–Metabolomic Study
Source: Metabolites. 2025 May 2;15(5):305. doi: 10.3390/metabo15050305 (PMC12113284; doi:10.3390/metabo15050305)
Supplement: Supplementary file 1 [file metabolites-15-00305-s001.zip › metabolites-3580814-supplementary.pdf]

Brown Algae from San Andres Island, SW Caribbean: An NMR-Metabolomic Study

Felipe de la Roche[1], Sara P. Abril[1], Lady J. Sepulveda[1], Anderson Piza[1], Leonardo Castellanos[1], Natalia Rincón[2], Mónica Puyana[2], Freddy A. Ramos[1]*.

[1]1Universidad Nacional de Colombia-Sede Bogotá, Facultad de Ciencias, Departamento de Química, Carrera 30 # 45-03, Bogotá DC, Colombia.; fde@unal.edu.co (F.d.R); spabrilp@unal.edu.co (S.P.A); lysepulvedas@unal.edu.co (L.Y.S); aepizas@unal.edu.co (A.P); lcastellanosh@unal.edu.co (L.C.); framosr@unal.edu.co (F. A. R)

[2]Universidad Jorge Tadeo Lozano, Departamento de Ciencias Biológicas y Ambientales, Carrera 4 # 22-61, Bogotá DC, Colombia.; marthan.rincon@utadeo.edu.co; monica.puyana@utadeo.edu.co (M.P)

*Correspondence: faramosr@unal.edu.co; Tel.: +57-1-316-5000 (ext. 14451)

Table S1. List of the species used for the metabolomic analysis; organic extracts were made with individual thali of each brown algae.

Code	Species	Collection Date	Growth Substrate
15A	*S. zonale*	January 2018	Dead coral
15B	*S. zonale*	January 2018	Dead coral
15C	*S. zonale*	January 2018	Dead coral
16A	*D. delicatula*	January 2018	Dead coral
16B	*D. delicatula*	January 2018	Dead coral
16C	*D. delicatula*	January 2018	Dead coral
17A	*D. delicatula*	January 2018	Dead coral
17B	*D. delicatula*	January 2018	Dead coral
17C	*D. delicatula*	January 2018	Dead coral
18A	*D. delicatula*	January 2018	Sand
18B	*D. delicatula*	January 2018	Sand
18C	*D. delicatula*	January 2018	Sand
19A	*D. delicatula*	January 2018	Sand
19B	*D. delicatula*	January 2018	Sand
19C	*D. delicatula*	January 2018	Sand
20A	*D. delicatula*	January 2018	Sand
20B	*D. delicatula*	January 2018	Sand
20C	*D. delicatula*	January 2018	Sand
21A	*D. delicatula*	January 2018	Dead coral
21B	*D. delicatula*	January 2018	Dead coral
34A	*C. crispatus*	January 2019	Rock
34B	*C. crispatus*	January 2019	Rock
34C	*C. crispatus*	January 2019	Rock
35A	*P. gymnospora*	January 2019	Rock
35B	*P. gymnospora*	January 2019	Rock
35C	*P. gymnospora*	January 2019	Rock

37A	*P. gymnospora*	January 2019	Sand
37B	*P. gymnospora*	January 2019	Sand
37C	*P. gymnospora*	January 2019	Sand
38A	*C. crispatus*	January 2019	Dead coral
38B	*C. crispatus*	January 2019	Dead coral
38C	*C. crispatus*	January 2019	Dead coral
39A	*S. zonale*	January 2019	Dead coral
39B	*S. zonale*	January 2019	Dead coral
39C	*S. zonale*	January 2019	Dead coral
40A	*S. zonale*	January 2019	Rock
40B	*S. zonale*	January 2019	Rock
40C	*S. zonale*	January 2019	Rock
41A	*D. delicatula*	January 2019	Sand
41B	*D. delicatula*	January 2019	Sand
41C	*D. delicatula*	January 2019	Sand
42A	*C. crispatus*	January 2019	Dead coral
42B	*C. crispatus*	January 2019	Dead coral
42C	*C. crispatus*	January 2019	Dead coral
43A	*C. crispatus*	January 2019	Rock
43B	*C. crispatus*	January 2019	Rock
43C	*C. crispatus*	January 2019	Rock
44A	*D. delicatula*	January 2019	Dead coral
44B	*D. delicatula*	January 2019	Dead coral
44C	*D. delicatula*	January 2019	Dead coral
45A	*C. crispatus*	January 2019	Rock
45B	*C. crispatus*	January 2019	Rock
45C	*C. crispatus*	January 2019	Rock
49A	*C. crispatus*	May 2019	Rock
49B	*C. crispatus*	May 2019	Rock
49C	*C. crispatus*	May 2019	Rock
50A	*C. crispatus*	May 2019	Rock
50B	*C. crispatus*	May 2019	Rock
50C	*C. crispatus*	May 2019	Rock
51A	*C. crispatus*	May 2019	Coral
51B	*C. crispatus*	May 2019	Coral
51C	*C. crispatus*	May 2019	Coral
52A	*S. zonale*	May 2019	Dead coral
52B	*S. zonale*	May 2019	Dead coral
53A	*S. zonale*	May 2019	Dead coral
53B	*S. zonale*	May 2019	Rock
53C	*S. zonale*	May 2019	Rock
54A	*S. zonale*	May 2019	Rock
54B	*S. zonale*	May 2019	Rock
54C	*S. zonale*	May 2019	Rock
55A	*P. gymnospora*	May 2019	Sand

55B	*P. gymnospora*	May 2019	Sand
55C	*P. gymnospora*	May 2019	Sand
56A	*P. gymnospora*	May 2019	Rock
56B	*P. gymnospora*	May 2019	Rock
56C	*P. gymnospora*	May 2019	Rock
57A	*P. gymnospora*	May 2019	Rock
57B	*P. gymnospora*	May 2019	Rock
57C	*P. gymnospora*	May 2019	Rock
58A	*D. delicatula*	May 2019	Sand
58B	*D. delicatula*	May 2019	Sand
58C	*D. delicatula*	May 2019	Sand
59A	*D. delicatula*	May 2019	Sand
59B	*D. delicatula*	May 2019	Sand
59C	*D. delicatula*	May 2019	Sand
60A	*D. delicatula*	May 2019	Dead coral
60B	*D. delicatula*	May 2019	Dead coral
60C	*D. delicatula*	May 2019	Dead coral
61A	*Dictyota* spp.	October 2019	Rock
61B	*Dictyota* spp.	October 2019	Rock
61C	*Dictyota* spp.	October 2019	Rock
62A	*Dictyota* spp.	October 2019	Rock
62B	*Dictyota* spp.	October 2019	Rock
63C	*Dictyota* spp.	October 2019	Rock

^{1}H-NMR species comparison metabolomic experiment

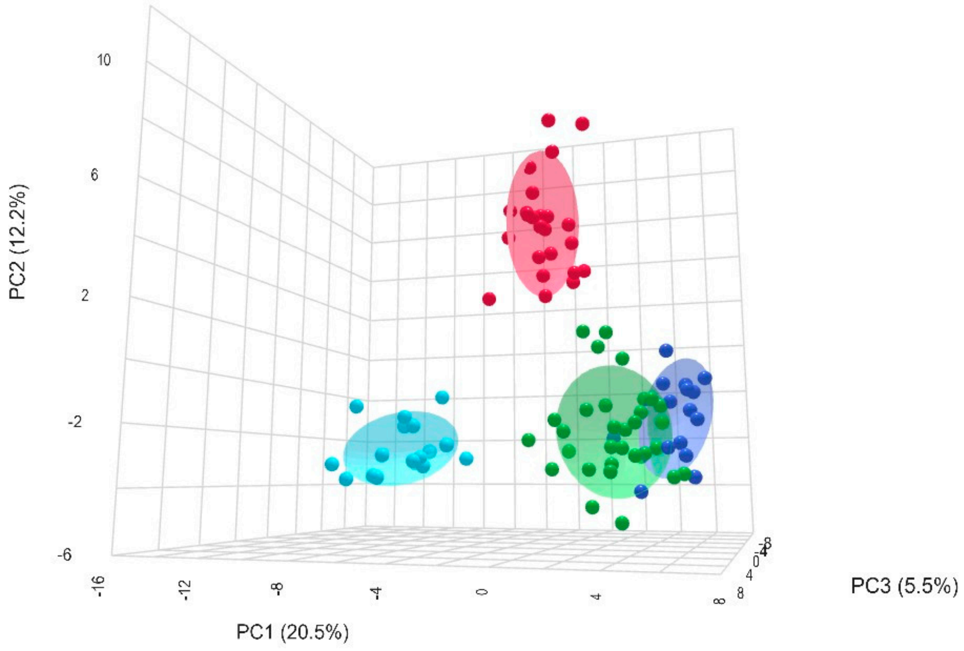

Figure S1. Principal component analysis scores plot (n=88) for *Canistrocarpus crispatus* (red), *Stypopodium zonale* (light blue), *Dictyopteris delicatula* (green), *Padina gymnospora* (Dark Blue) using component 1 (20.5%), component 2 (12.2%) and component 3 (5.5%) in the axes.

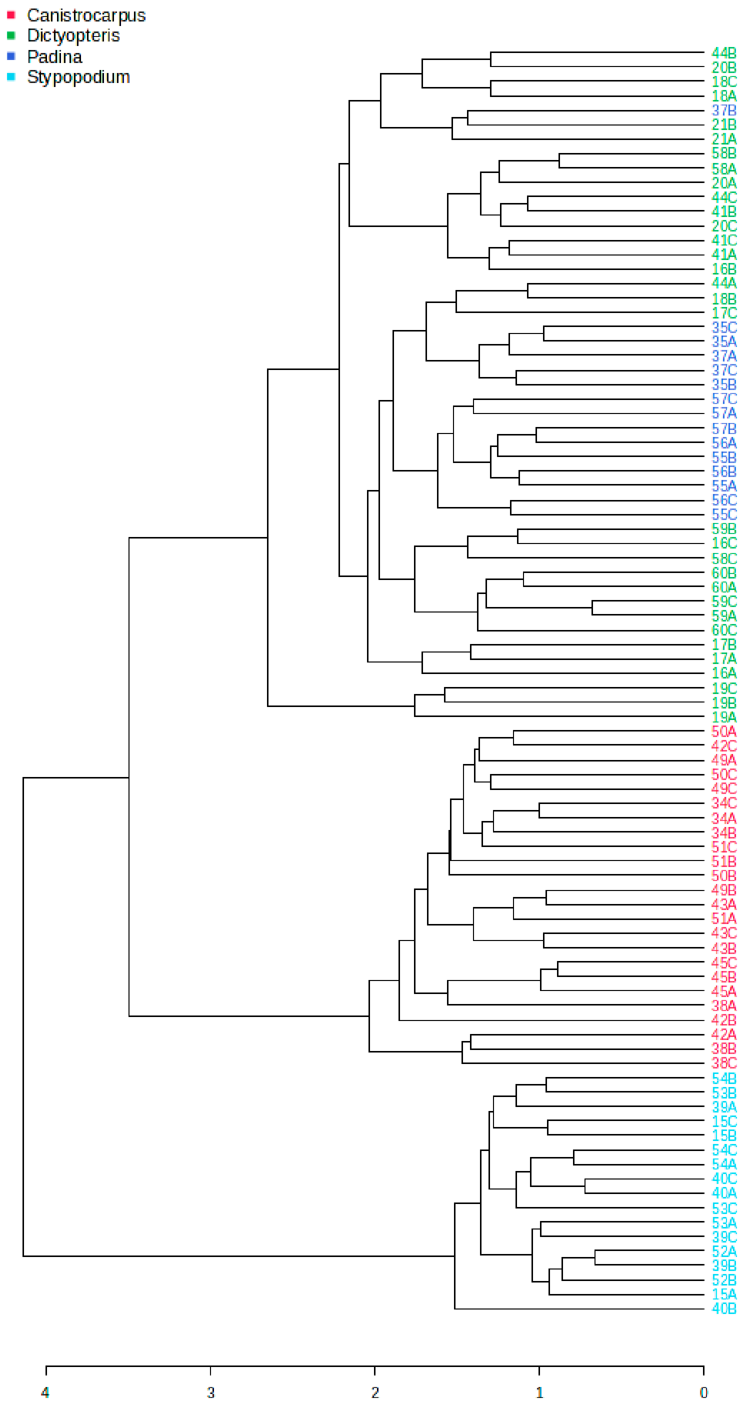

Figure S2. Hierarchical cluster analysis (HCA) dendrogram (n=88) generated from ^{1}H-NMR spectra of organic fractions of brown algae collected in shallow environments north of the island of San Andrés. *Canistrocarpus crispatus* (red), *Stypopodium zonale* (light blue),

Dictyopteris delicatula (green), *Padina gymnospora* (Dark Blue) Pearson's correlation was used as a distance measure and grouping was performed using the average algorithm.

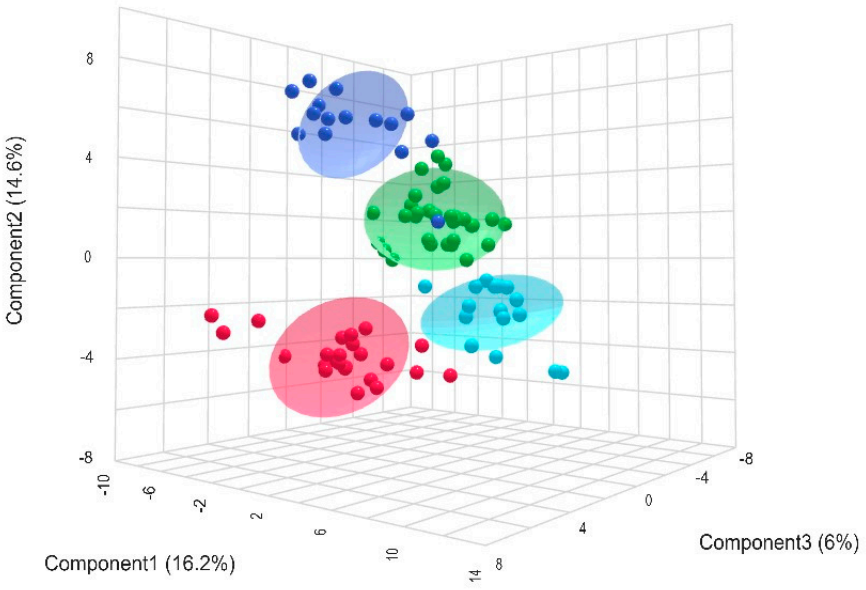

Figure S3. Partial least discriminant analysis PLS-DA scores plots (n=88) for *Canistrocarpus crispatus* (red), *Stypopodium zonale* (light blue), *Dictyopteris delicatula* (green), *Padina gymnospora* (Dark Blue) using component 1 (16.2%), component 2 (14.6%) and component 3 (6.0%) in the axes. R^2 0.96% Q^2:0.87%.

Table S2. Cross validation of the model generated in the PLS-DA of the ^{1}H-NMR spectra of organic fractions of brown algae collected in shallow environments north of the island of San Andrés including the R^2 and Q^2 statistics for components 1, 2 and 3.

**Measure**	**1 comp**	**2 comps**	**3 comps**
**Accuracy**	0.66	0.83	0.92
**R^2**	0.75	0.87	0.93
**Q^2**	0.69	0.81	0.87

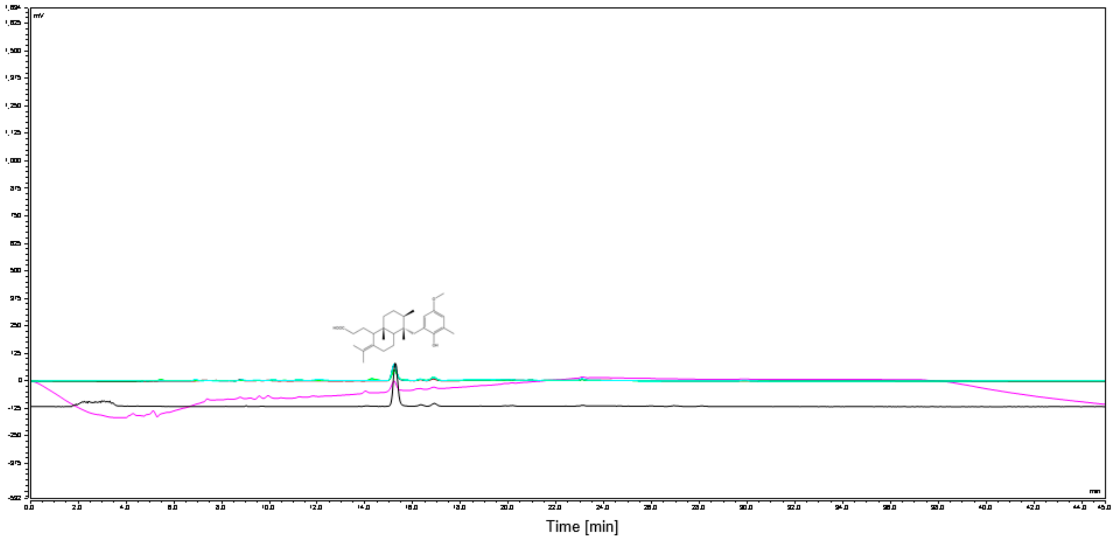

Figure S4. HPLC chromatogram of the organic extract from *Stypopodium zonale*, acquired using DAD (Pink 210 nm, Red 254 nm, Green 300 nm, Blue 366 nm) and ELSD (Black) detectors, shows an intense peak at 15 minutes corresponding to atomaric acid **(1)**.

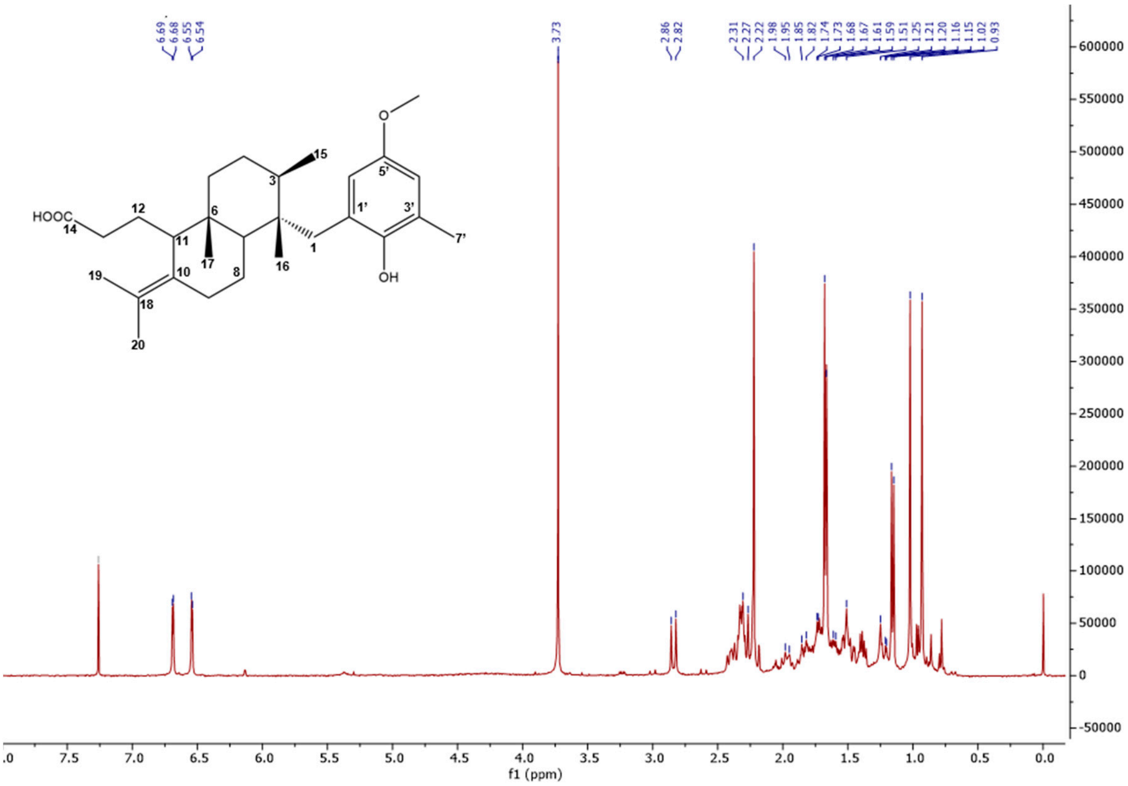

Figure S5. ^{1}H-NMR spectrum ($CDCl_3$, 400MHz) of atomaric acid **(1)** from *S. zonale*.

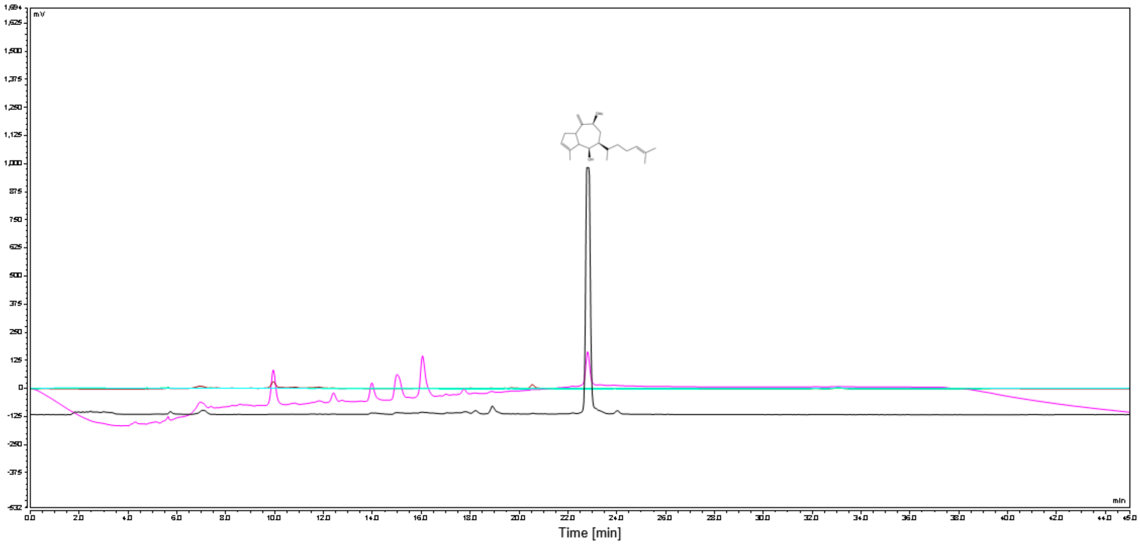

Figure S6. HPLC chromatogram of the organic fraction F3 from *Canistrocarpus crispatus*, acquired using DAD (Pink 210 nm, Red 254 nm, Green 300 nm, Blue 366 nm) and ELSD (Black) detectors, shows a peak at 21 minutes corresponding to dictyol B acetate **(2)**.

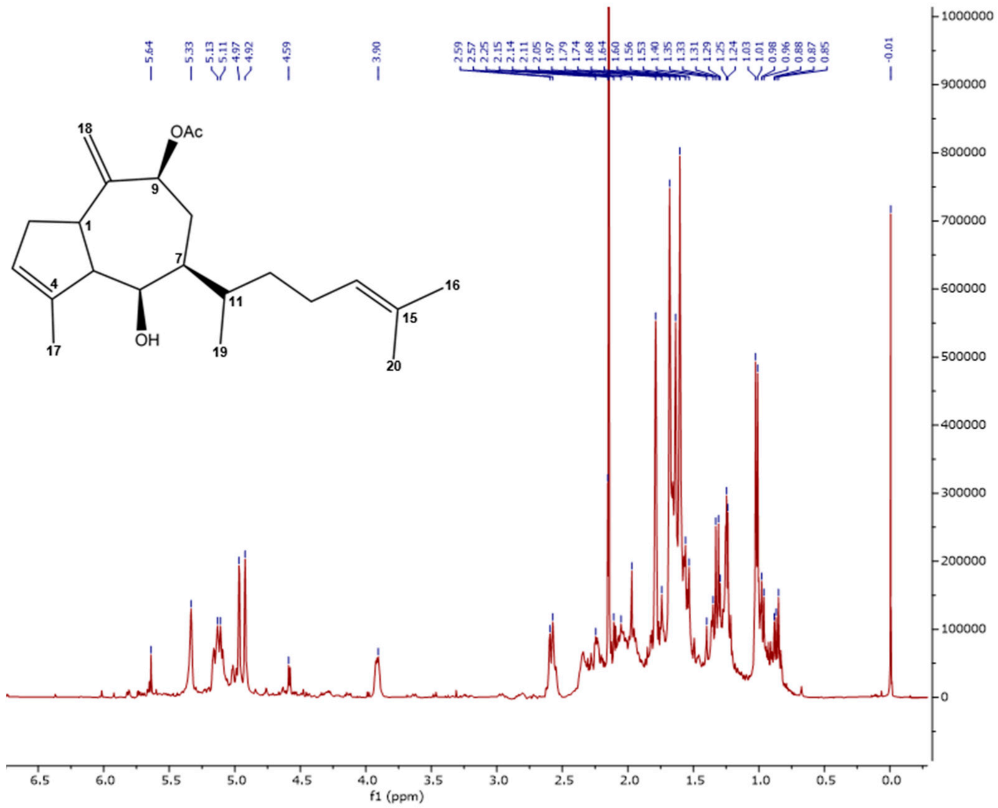

Figure S7. ^{1}H-NMR spectrum ($CDCl_3$, 400MHz) of dictyol B acetate **(2)** isolated from *C. crispatus*.

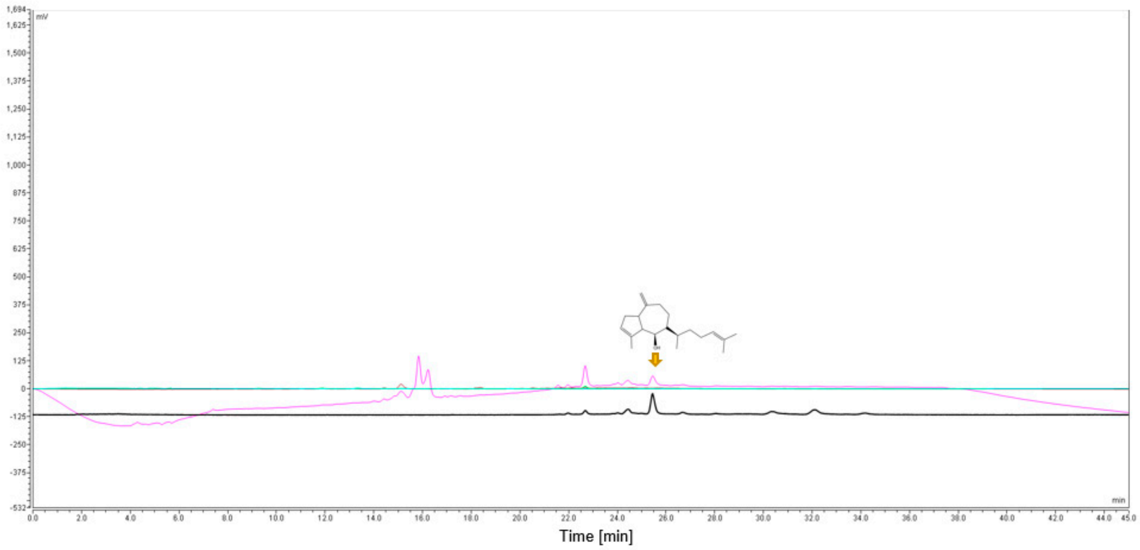

Figure S8. HPLC chromatogram of the organic fraction F2 from *Canistrocarpus crispatus*, acquired using DAD (Pink 210 nm, Red 254 nm, Green 300 nm, Blue 366 nm) and ELSD (Black) detectors, shows a peak at 25 minutes corresponding to pachydictyol A.

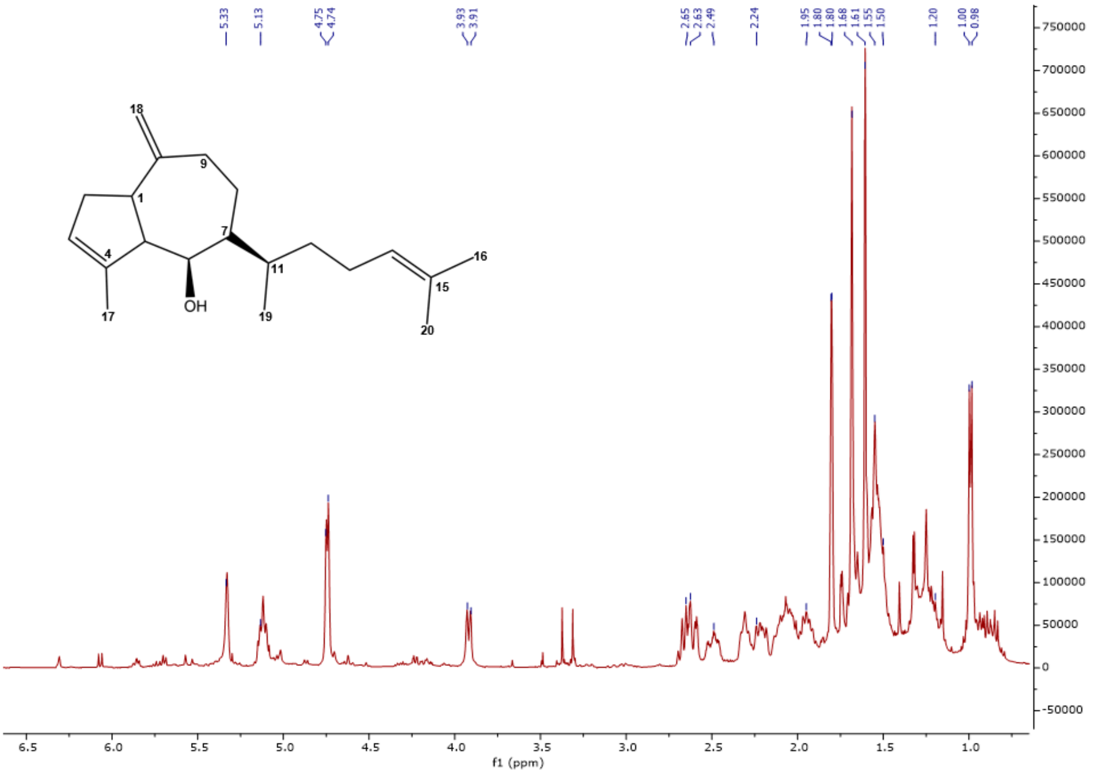

Figure S9. ^{1}H-NMR spectrum ($CDCl_3$, 400MHz) of pachydictyol A isolated from *C. crispatus*.

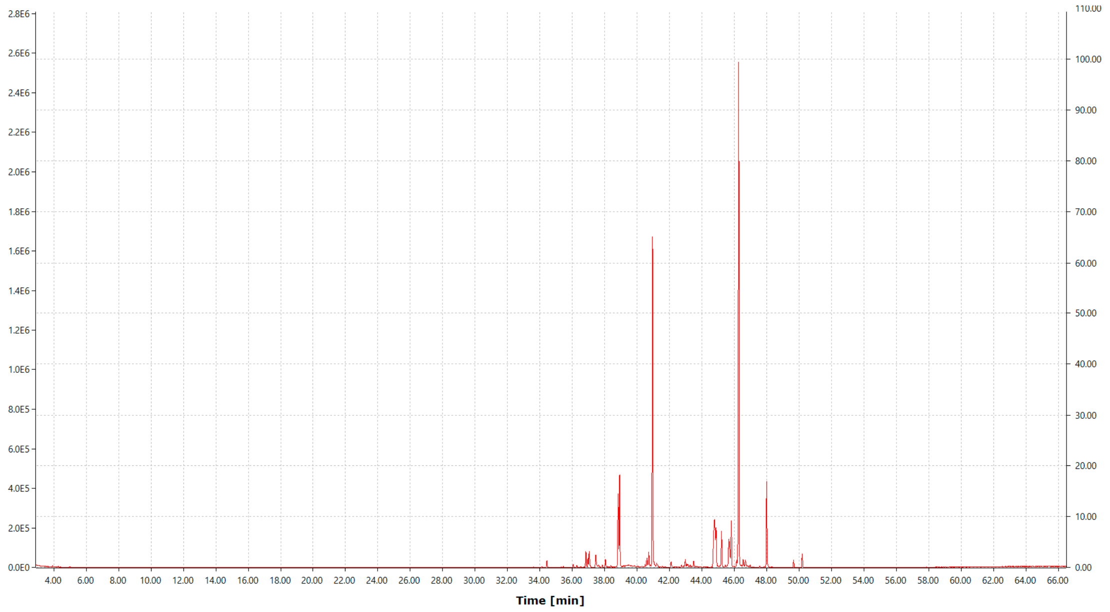

Figure S10. GC-MS chromatogram of the organic fraction F3 from *Canistrocarpus crispatus* shows a peak at 46 minutes identified as dictyol B acetate.

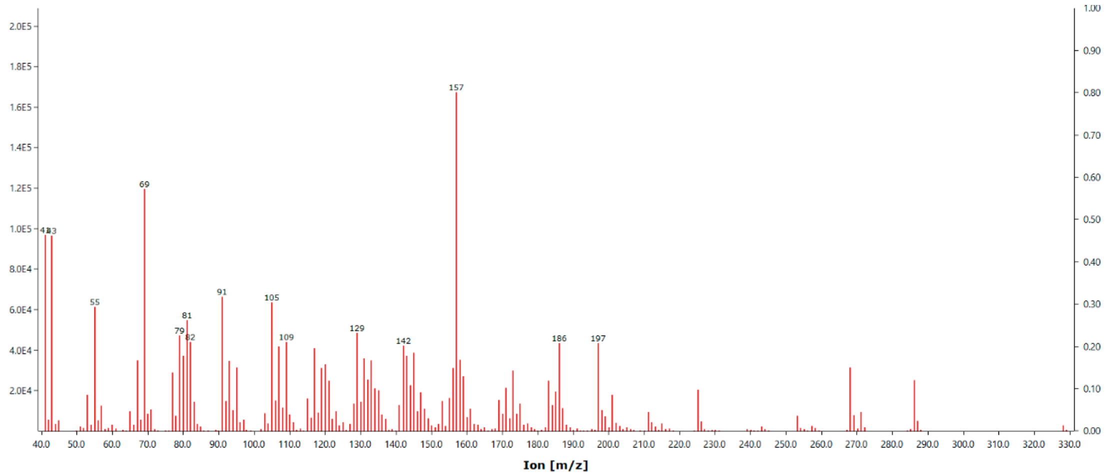

Figure S11. Mass spectrum obtained by GC-MS (EI, 70 eV) of compound (2) dictyol B acetate.

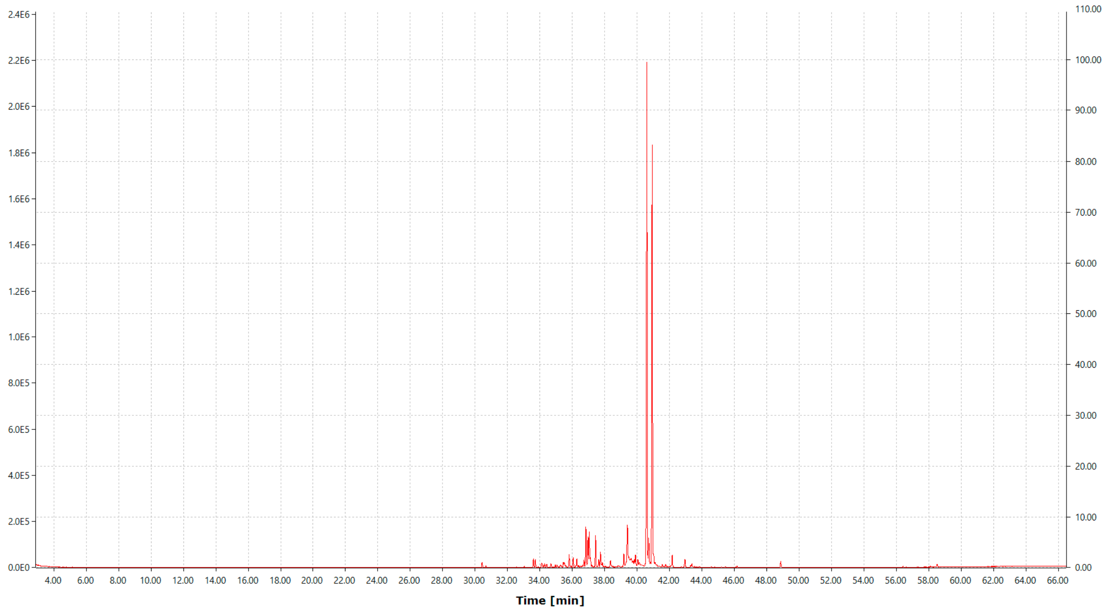

Figure S12. GC-MS chromatogram from the organic fraction F2 from *Canistrocarpus crispatus*, shows a peak at min 41 identified as pachydictyol A.

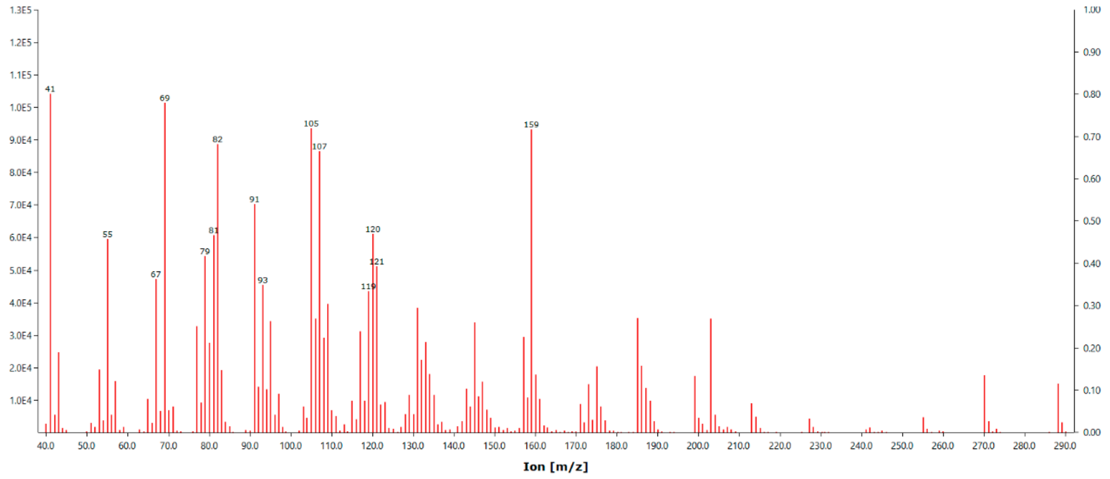

Figure S13. Mass spectrum obtained by GC-MS (EI, 70 eV) of pachydictyol A.

Table S3. ^{1}H ($CDCl_3$, 400 MHz) and ^{13}C ($CDCl_3$, 100 MHz) chemical shifts for compounds atomaric acid (1) isolated from *S. zonale,* also compounds dictyol B acetate (2) and pachydictyol A isolated from *C. crispatus*.

	**atomaric acid (1)**		**dictyol B acetate (2)**		**pachydictyol A**	
**# C**	**^{1}H δ ppm (m, *J* Hz)**	**^{13}C δ ppm**	**^{1}H δ ppm (m, *J* Hz)**	**^{13}C δ ppm**	**^{1}H δ ppm (m, *J* Hz)**	**^{13}C δ ppm**
**1**	2,24 (d, J=14, 1H), 2,86 (d, J=14, 1H)	35,4	2.60* (d, J=9.3Hz, 1H)	43.1	2.65 (m, 1H)	46.2
**2**	-	40,9	2.60* (m, 1H), 2.26 (m, 1H)	33.8	2.49 (m, 1H), 2.21 (m, 1H)	33.9
**3**	1,73 (m, 1H)	35,4	5.33 (m, 1H)	124.0	5.33 (m, 1H)	124.1
**4**	1,21 (m, 1H), 1,82 (m, 1H)	25,5	-	140.9	-	141.5
**5**	1,51 (m, 1H)	36,6	2,37 (m, 1H)	61.1	2.31 (m, 1H)	60.4
**6**	-	38,9	3.91 (dd, J= 8.2, 3.7 Hz, 1H)	74.6	3.92 (d, J=7.79)	75.2
**7**	1,38 (m, 1H)	42,0	1.68** (m, 1H)	44.0	1.55 (m, 1H)	47.9
**8**	1,73 (m, 1H), 1,52 (m, 1H)	22,4	1.82 (m, 1H), 1.67 (m, 1H)	30.3	1.50 (m, 2H)	23.6
**9**	1,95 (m, 1H), 2,37 (m, 1H)	23,5	5.17*** (m, 1H)	77.4	2.62 (m, 1H) 2.10 (m, 1H)	40.7
**10**	-	132,9	-	149.6	-	152.7
**11**	2,33(m, 1H)	53,2	1.61 (m, 1H)	34.8	1.20 (m, 1H)	34.8
**12**	1,81 (m, 1H), 1,61 (m, 1H)	25,1	1.54 (m, 1H), 1.26 (m, 1H)	35.0	2.24 (m, 1H) 1.53 (m, 1H)	35.1
**13**	2,29 (m, 1H)	33,0	2.07(m, 1H) 1.95(m, 1H)	25.7	2.04 (m, 1H), 1.95 (m, 1H)	25.7
**14**	-	180,4	5.14*** (m, 1H)	124.6	5.13 (m, 1H)	124.8
**15**	1,16 (d, J=6,9, 3H)		-	131.9	-	131.7
**16**	0,93 (s, 3H)	20,8	1.68** (s, 3H)	25.9	1.68 (brs, 3H)	25.8
**17**	1,03 (s, 3H)	17,9	1.79 (s, 3H)	15.8	1.80 (brs, 3H)	16.0
**18**	-	123,4	4.97 (br,1H), 4.92 (br, 1H)	104.8	4.74 (brs, 1H) 4.75(brs, 1H)	107.2
**19**	1,68 (m, 3H)	20,4	1.01 (d, J=6.14 Hz, 3H)	17.5	0.99 (d, J=6.0 Hz, 3H)	17.6
**20**	1,66 (s, 3H)	20,4	1.60 (s, 3H)	17.8	1.61 (s, 3H)	17.8
**1′**	-	127,1	-	-	-	

**2′**	-	146,9	-	-	-	-
**3′**	-	124,1	-	-	-	-
**4′**	6,54 (d, J=2,6, 1H)	113,6	-	-	-	-
**5′**	-	152,5	-	-	-	-
**6′**	6,69 (d, J=3,0, 1H)	114,5	-	-	-	-
**7′**	2,22 (s, 3H)	16,5	-	-	-	-
**Ome5′**	3,73(s, 3H)	55,5	-	-	-	-
**COMe**	-	-	-	170.2	-	-
**COMe**	-	-	2.14 (s, 3H)	21.4	-	-

*Overlapping signals, ** overlapping signals, *** overlapping signals.

Effect of collection time and growth substrate on the metabolic profiles of brown algae species

Growth substrate

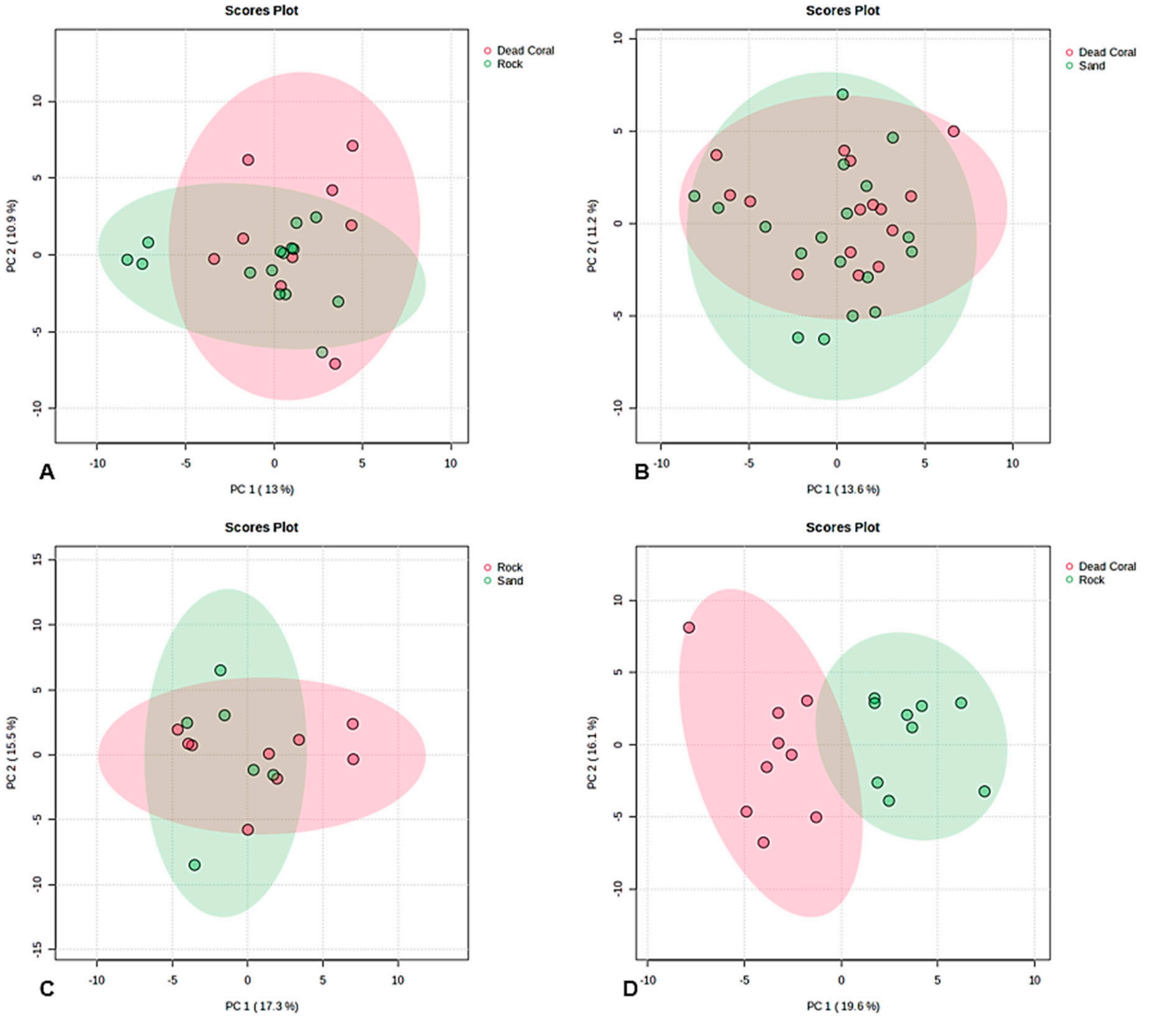

Figure S14. Principal component analysis PCA scores plot comparing brown algae species ^{1}H organic extracts between growth substrate (Sand, dead coral and rock). **A:** *C. crispatus*, **B:** *D. delicatula*, **C:** *P. gymonspora*, **D:** *S. zonale*.

Table S4. Cross validation of the model generated in the PLS-DA of the ^{1}H-NMR spectra of organic fractions *S. zonale* collected in shallow environments (death coral and rock) north of the island of San Andrés including the R^2 and Q^2 statistics for components 1, 2, 3.

Measure	1 comp	2 comps	3 comps
Accuracy	1.0	1.0	1.0
R^2	0.88	0.96	0.99
Q^2	0.70	0.66	0.62

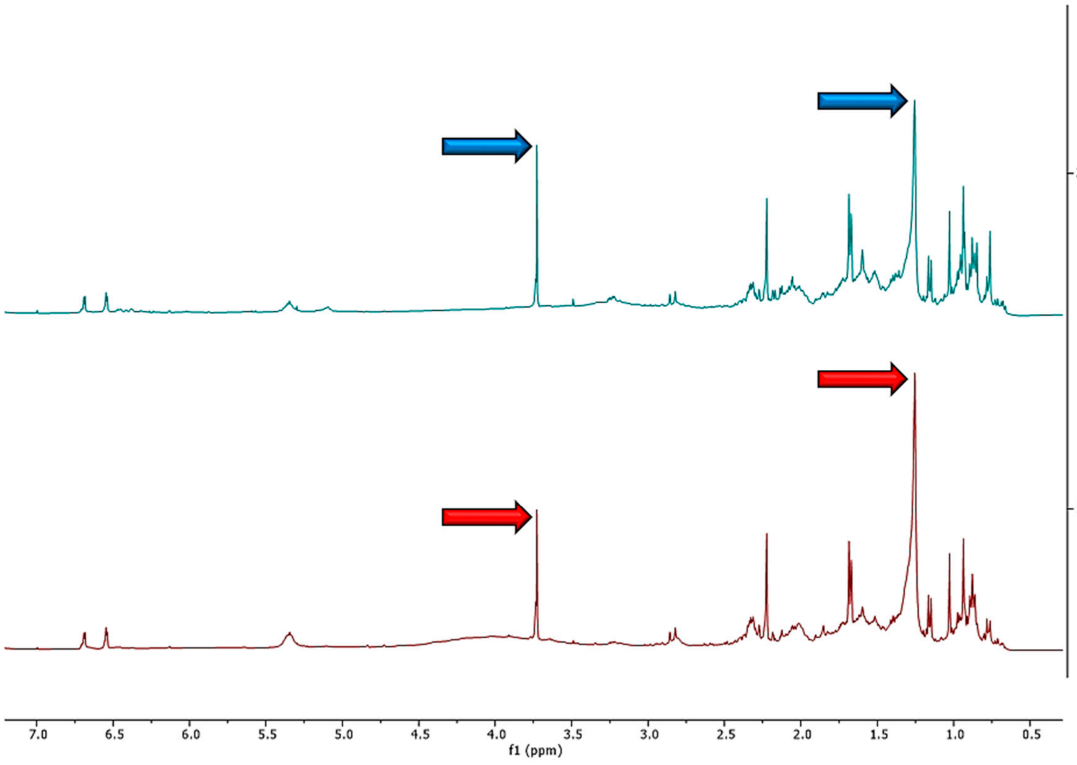

Figure S15. Overlapping ^{1}H-NMR spectra ($CDCl_3$, 400MHz) of organic extracts from *S. zonale* collected in dead coral (Blue) and rock (Red).

Collection date

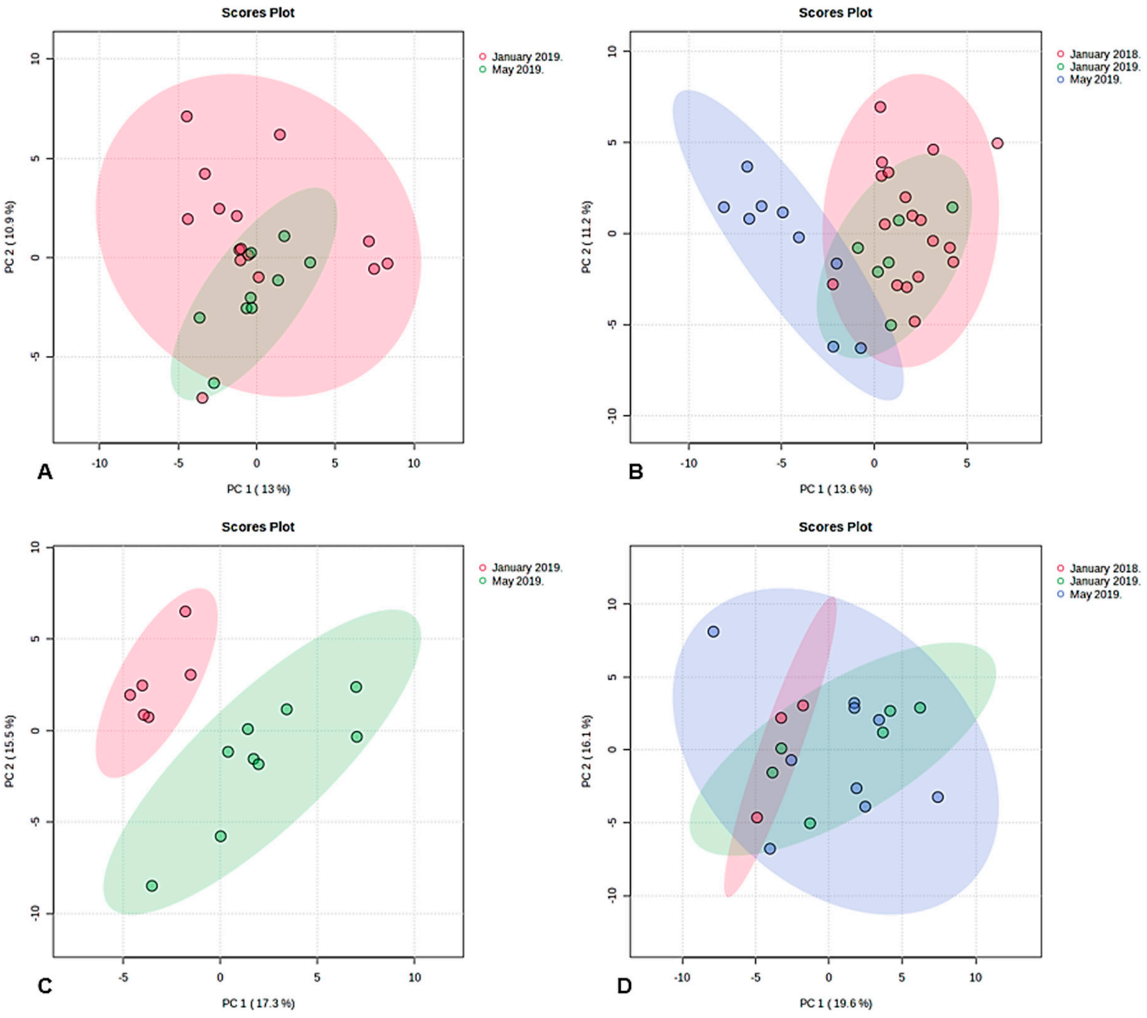

Figure S16. Principal component analysis PCA scores plot comparing brown algae species ^{1}H organic extracts between collection dates (May 2018, January 2019 and May 2019). **A:** *C. crispatus*, **B:** *D. delicatula*, **C:** *P. gymonspora*, **D:** *S. zonale*.

Table S5. Cross validation of the model generated in the PLS-DA of the ^{1}H-NMR spectra of organic fractions of *P. gymnospora* collected in shallow environments north of the island of San Andrés in different collection dates including the R^2 and Q^2 statistics for components 1, 2, 3.

**Measure**	**1 comp**	**2 comps**	**3 comps**
Accuracy	1.0	1.0	1.0
R^2	0.94	0.98	0.99
Q^2	0.75	0.77	0.77

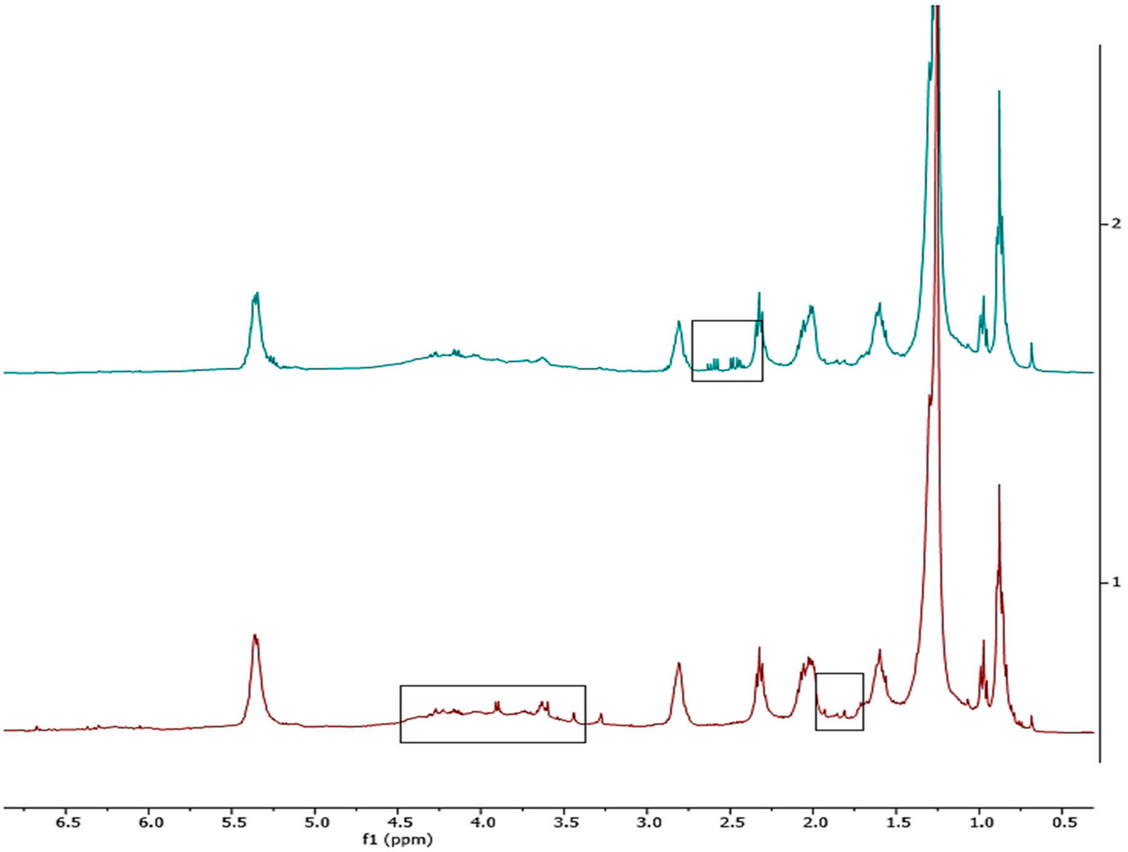

Figure S17. Overlapping ^{1}H-NMR spectra ($CDCl_3$, 400MHz) of organic extracts from spectra of *P. gymnospora* collected in January (Blue) and May 2019 (Red).

Juvenile *Dictyota* spp. metabolic profile

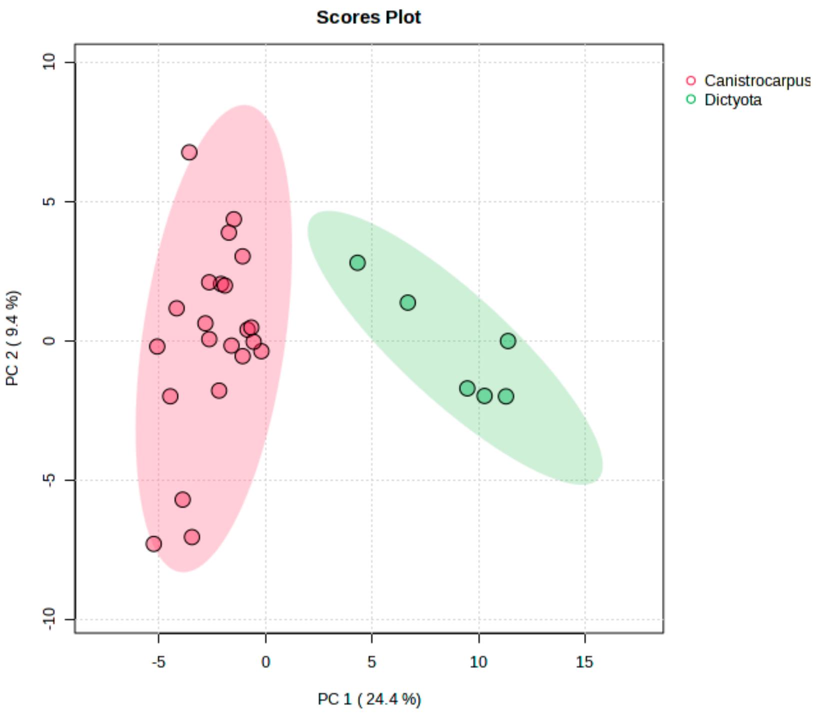

Figure S18. Principal component analysis scores plots (n=28) comparing juvenile *Dictyota* spp. and *C. crispatus* samples using component 1 (24.4%) and component 2 (9.4) in the axes.

Table S6. Cross validation of the model generated in the PLS-DA of the ^{1}H-NMR spectra of organic fractions of brown algae collected in shallow environments north of the island of San Andrés including the R^2 and Q^2 statistics for components 1, 2, 3.

Measure	1 comp	2 comps	3 comps
Accuracy	1.0	1.0	1.0
R^2	0.91	0.98	0.99
Q^2	0.86	0.90	0.91

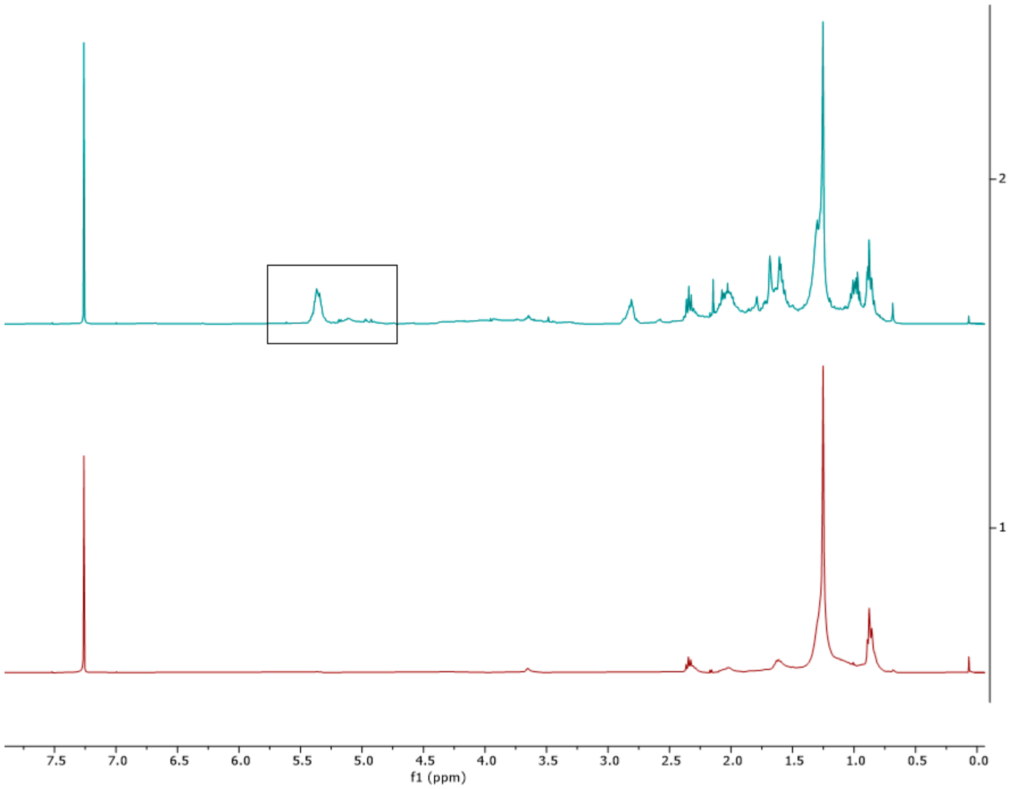

.

Figure S19. Overlapping ^{1}H-NMR spectra ($CDCl_3$, 400MHz) of organic extracts from spectra of *C. crispatus* (Blue) and *Dictyota* spp. (Red).
